# Immunosuppressive *Yersinia* Effector YopM Binds DEAD Box Helicase DDX3 to Control Ribosomal S6 Kinase in the Nucleus of Host Cells

**DOI:** 10.1371/journal.ppat.1005660

**Published:** 2016-06-14

**Authors:** Laura Berneking, Marie Schnapp, Andreas Rumm, Claudia Trasak, Klaus Ruckdeschel, Malik Alawi, Adam Grundhoff, Alexey G. Kikhney, Friedrich Koch-Nolte, Friedrich Buck, Markus Perbandt, Christian Betzel, Dmitri I. Svergun, Moritz Hentschke, Martin Aepfelbacher

**Affiliations:** 1 Institute of Medical Microbiology, Virology and Hygiene, University Medical Center Hamburg-Eppendorf (UKE), Hamburg, Germany; 2 Bioinformatics Core, University Medical Center Hamburg-Eppendorf, Hamburg, Germany; 3 Heinrich-Pette-Institute (HPI), Leibniz Institute for Experimental Virology, Research Group Virus Genomics, Hamburg, Germany; 4 European Molecular Biology Laboratory (EMBL), Hamburg Outstation, Hamburg, Germany; 5 Institute of Immunology, University Medical Center, Hamburg, Germany; 6 Institute of Clinical Chemistry, University Medical Center, Hamburg, Germany; 7 Institute of Biochemistry and Molecular Biology, University of Hamburg, Laboratory of Structural Biology of Infection and Inflammation, Hamburg, Germany; 8 The Hamburg Centre for Ultrafast Imaging, Hamburg, Germany; Purdue University, UNITED STATES

## Abstract

*Yersinia* outer protein M (YopM) is a crucial immunosuppressive effector of the plaque agent *Yersinia pestis* and other pathogenic *Yersinia* species. YopM enters the nucleus of host cells but neither the mechanisms governing its nucleocytoplasmic shuttling nor its intranuclear activities are known. Here we identify the DEAD-box helicase 3 (DDX3) as a novel interaction partner of *Y*. *enterocolitica* YopM and present the three-dimensional structure of a YopM:DDX3 complex. Knockdown of DDX3 or inhibition of the exportin chromosomal maintenance 1 (CRM1) increased the nuclear level of YopM suggesting that YopM exploits DDX3 to exit the nucleus via the CRM1 export pathway. Increased nuclear YopM levels caused enhanced phosphorylation of Ribosomal S6 Kinase 1 (RSK1) in the nucleus. In *Y*. *enterocolitica* infected primary human macrophages YopM increased the level of Interleukin-10 (IL-10) mRNA and this effect required interaction of YopM with RSK and was enhanced by blocking YopM's nuclear export. We propose that the DDX3/CRM1 mediated nucleocytoplasmic shuttling of YopM determines the extent of phosphorylation of RSK in the nucleus to control transcription of immunosuppressive cytokines.

## Author Summary

Diarrheagenic bacteria like *Salmonella*, *Shigella* and *Yersinia* employ a molecular injection apparatus (termed type III secretion system) to introduce toxins into host immune cells. This strategy strongly suppresses the immune response and promotes infection. Once within the host cell the bacterial toxins distribute to different subcellular compartments including the nucleus. The major immunosuppressive protein YopM from pathogenic *Yersinia* species, which has homologues in *Salmonella* and *Shigella*, enters the nucleus of host cells. So far it has been unclear what regulates the shuttling of YopM in and out of the nucleus and which molecular activities related to immunosuppression YopM may exert in the nucleus. Here we identify a protein named DDX3 that transports YopM out of the nucleus and thereby adjusts the nuclear level of YopM. We also show that the nuclear level of YopM controls nuclear phosphorylation/activation of the multifunctional kinase RSK1, a known regulator of gene transcription. Furthermore, association of YopM with RSK was required for increased expression of the immunosuppressive cytokine IL-10. Thus we describe a mechanism by which a bacterial virulence factor shuttles between cytosol and nucleus and demonstrate that this influences transcription of immune mediator genes.

## Introduction

*Yersinia* outer protein M (YopM) is a major virulence factor of the three pathogenic *Yersinia* species, *Y*. *pestis*, *Y*. *pseudotuberculosis* and *Y*. *enterocolitica* [1, 2]. Together with up to six other effector proteins YopM is translocated into host cells by the bacterial type three secretion apparatusand significantly contributes to the *Yersinia* infection strategy [3–5]. In mouse infection models *Yersinia* YopM mutants showed a reduction of virulence by up to 5 orders of magnitude. The magnitude of the YopM effect depended on the *Yersinia* strains and mouse models as well as the infection routes employed [4, 6–8].

YopM was found to enter the nucleus of mammalian- and yeast cells whereby the nuclear levels of YopM varied between individual cells and significant amounts of the protein also reside in the cytoplasm [9–13]. YopM proteins from different *Yersinia* strains display apparent molecular weights from 41 to 55 Kilodalton (kDa) in SDS PAGE [2]. This heterogeneity arises from a variable number of leucin rich repeats (LRRs), ranging from 13 in YopM from *Y*. *enterocolitica* 8081 to 21 in YopM from *Y*. *pseudotuberculosis* IP32953 [14]. *Y*. *pestis* and *Y*. *enterocolitica* YopM appear to traffic to the nucleus via a vesicle-associated pathway [11, 15]. Attempts to identify a conventional nuclear localization signal (NLS) in YopM were unsuccessful but instead both, the N-terminal three LRRs and the C-terminal tail of YopM were found to be sufficient but not essential for nuclear targeting. The C-terminal tail was also suggested to function as a non-conventional bipartite NLS [9, 12]. An importin (karyopherin) mediating nuclear import of YopM has not been identified to date [9]. Further, neither the molecular mechanisms regulating nucleocytoplasmic shuttling of YopM nor any activity of nuclear localized YopM have been reported.

The individual LRRs in YopM consist of 20- or 22 amino acids and together form a LRR-domain, which makes up the central and largest part of the protein. The sequences of the first 3 and the last 2–4 LRRs of the different LRR-domains are highly conserved whereas the remaining LRRs are more divergent [14]. In comparison, the N-terminus with the secretion/translocation region followed by two α-helices that are thought to aid LRR folding and the unstructured C-terminal tail are highly homologous in all YopM isoforms [16]. YopM of *Y*. *pestis* 195/P was crystallized and presented as a twisted horseshoe-like structure formed by an LRR-domain consisting of 15 LRRs [16]. The N-terminal 33 amino acids and the C-terminal 24 amino acids of the crystallized protein could not be resolved. The concave side of the LRR-domain is made up by parallel β-sheets and exposes numerous amino acid side chains, which in homologous proteins have been proposed to represent the binding surface for eukaryotic partners [17, 18]. Within the crystals four YopM monomers formed a tetrameric superhelix encompassing a hollow cylinder with an inner diameter of 35 Å [16]. It has so far been unclear in which form YopM exists in solution and how interaction with any of its eukaryotic binding partners is organized on the structural level.

YopM isoforms of all three pathogenic *Yersinia* species were found to associate with the serine/threonine kinases RSK1 (p90 ribosomal S6 kinase 1; MAPKAP-K1) and PKN2 (protein kinase N2; protein kinase C related kinase2/PRK2) [7, 8, 19, 20]. Formation of a complex containing YopM, RSK1 (or RSK2, -3, or -4; [20]) and PKN2 (or PKN1, or -3; [20]) induced hyperphosphorylation and activation of RSK by shielding it from dephosphorylation by protein phosphatases [20]. In this complex PKN, which is not a physiological RSK substrate, was proposed to become phosphorylated by RSK [19]. Members of the RSK family are downstream effectors of the extracellular signal-regulated kinases 1/2 (ERK 1/2) and translocate to the nucleus upon activation where they control gene expression [21]. The C-terminal 6 amino acids of YopM were found to be required for the interaction with RSK1 whereas the LRR domain was necessary for interaction with PKN2 [7, 8]. YopM mutants that were unable to bind RSK1 still localized to the nucleus excluding a nuclear import function of RSK1 [7]. At present no downstream activity of the YopM:RSK:PKN complex has been reported, albeit YopM mutants that lost binding to RSK or PKN were severely attenuated in mouse virulence [7, 8].

In *Y*. *pestis* infected mice a tagged YopM was detected in monocytes/macrophages, neutrophils and dendritic cells but not in T- or B-cells [22]. Consistent with this, *Y*. *pestis* YopM inhibited the recruitment of neutrophils, monocytes and inflammatory dendritic cells to the spleen at 2 days post infection [23, 24]. Furthermore, YopM from different *Yersinia* strains reduced the production of proinflammatory cytokines (i.e. Interferon–γ (IFN-γ), Interleukin-1β (IL-1β), Tumor Necrosis Factor-α (TNF-α), Interleukin-15 (IL-15)) in mice at 1–4 days post infection [8, 25]. Recently, YopM isoforms from *Y*. *pestis* and *Y*. *pseudotuberculosis* were found to inhibit IL-1β production in primary mouse macrophages and macrophage cell lines [26–28]. In mice infected with *Y*. *pseudotuberculosis*, YopM increased the serum level of the immunosuppressive cytokine IL-10 and this was found to be important for virulence of the bacteria [8, 29]. Thus, innate immune cells and the network of inflammatory and antinflammatory cytokines produced by these cells during infection are considered to be prime targets of YopM.

Here we provide evidence that *Y*. *enterocolitica* WA314 YopM harboring 20 LRRs forms a 2:1 complex with the DDX3 and that the association with DDX3 mediates nuclear export of YopM via the exportin CRM1. We also demonstrate that nuclear YopM regulates nuclear RSK1 phosphorylation and that these YopM activities are associated with an increased expression of antiinflammatory immune response genes in human macrophages.

## Results

### DEAD box helicase DDX3 is a novel interaction partner of *Y*. *enterocolitica* YopM

**Fig 1:**
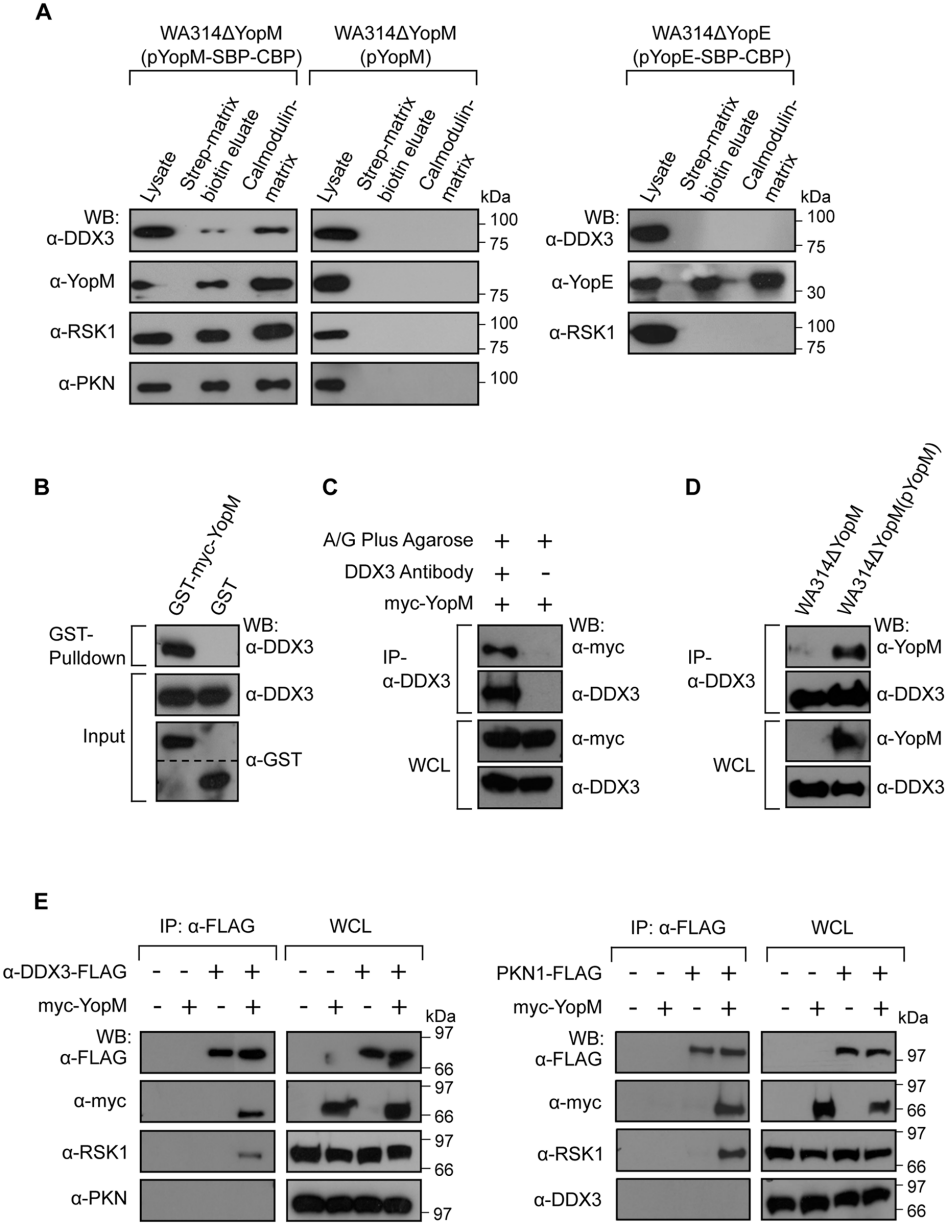
*Y*. *enterocolitica* YopM interacts with DDX3 in host cells. **A) Coprecipitation of bacterially translocated YopM-SBP-CBP with DDX3, RSK1 and PKN in J774A.1 cells**. J774A.1 cells were infected with WA314ΔYopM(pYopM-SBP-CBP), WA314ΔYopM(pYopM) or WA314ΔYopE(pYopE-SBP-CBP). Proteins eluting consecutively from streptavidin-sepharose (biotin elution) and calmodulin-sepharose (boiling in sample buffer) were analyzed by Western blot using indicated antibodies. **B) GST-YopM pull-down of endogenous DDX3**. GST-myc-YopM or GST expressed in HEK293T cells were precipitated with glutathione sepharose beads and analyzed by Western blot using indicated antibodies. **C) Myc-YopM co-immunoprecipitates with endogenous DDX3**. Endogenous DDX3 was immunoprecipitated in HEK293T cells expressing myc-YopM. Precipitates and whole cell lysates (WCL) were analyzed by Western blot using indicated antibodies. **D) Bacterially translocated YopM co-immunoprecipitates with endogenous DDX3**. Endogenous DDX3 was immunoprecipitated in HEK293T cells infected with WA314ΔYopM(pYopM) or WA314ΔYopM for 90 min. Precipitates and whole cell lysates (WCL) were analyzed by Western blot using indicated antibodies. **E) DDX3 and PKN mutually exclude each other in RSK1/YopM containing complexes**. HEK293T cells expressing either DDX3-FLAG (left panel) or PKN-FLAG (right panel) and where indicated myc-YopM were lysed and subjected to anti-FLAG immunoprecipitation. Precipitates and whole cell lysates (WCL) were analyzed by Western blot using indicated antibodies. In DDX3-FLAG immunoprecipitates, myc-YopM, RSK1 but no PKN and in PKN-FLAG immunoprecipitates myc-YopM, RSK1 but no DDX3 were detected.

To search for novel YopM binding partners we complemented a YopM deletion mutant of *Y*. *enterocolitica* serotype O:8, strain WA314 (WA314ΔYopM) with a tandem affinity purification (TAP)-tagged isogenic YopM, giving rise to WA314ΔYopM(pYopM-SBP-CBP) [20]. The TAP-tag consists of a streptavidin binding peptide (SBP) and a separate calmodulin binding peptide (CBP), which allows TAP-tagged proteins to be consecutively affinity precipitated on streptavidin-sepharose and calmodulin-sepharose affinity matrices [30]. To mimic physiological infection conditions, J774A.1 macrophages were infected with WA314ΔYopM(pYopM-SBP-CBP) to translocate YopM-SBP-CBP into the cells via the bacterial type three secretion apparatus. The translocated YopM-SBP-CBP was affinity precipitated from the infected cells and precipitates were analyzed by peptide mass fingerprinting. We previously reported that the well accepted YopM interaction partners RSK1 and PKN2 as well as their close homologues RSK2 and PKN1, respectively, coprecipitated with YopM-SBP-CBP [20]. This confirmed the principal suitability of the method to identify bona fide YopM binding partners. Another protein that coprecipitated with YopM-SBP-CBP in these experiments was the DEAD box RNA helicase DDX3 (S1 Table). To verify the specificity of the YopM interaction with DDX3 we infected J774A.1 cells with WA314ΔYopM(pYopM-SBP-CBP) and with two strains that either translocate an untagged YopM (WA314ΔYopM(pYopM)) or a TAP-tagged YopE (WA314ΔYopE(pYopE-SBP-CBP)). Lysates of the infected cells were subjected to consecutive SBP- and CBP affinity precipitation and the precipitates were tested for the presence of DDX3 by immunoblotting. DDX3 coprecipitated with YopM-SBP-CBP but not with YopE-SBP-CBP and was not present in precipitates obtained from the WA314ΔYopM(pYopM) infected cells (Fig 1A). In addition to DDX3, RSK1 and PKN coprecipitated with YopM-SBP-CBP (Fig 1A). Endogenous DDX3 also coprecipitated with glutathione S-transferase- (GST) and myc tagged-YopM (GST-myc-YopM) expressed in HEK293T cells (Fig 1B). Reciprocal experiments revealed that both, cellular expressed myc-YopM and bacterially translocated YopM coimmunoprecipitated with endogenous DDX3 in HEK293T cells (Fig 1C and 1D). Together these experiments show that YopM associates with DDX3 in *Yersinia* infected host cells.

In previous work it was demonstrated that YopM can form a ternary complex with RSK1 and PKN2. This was concluded because in immunoprecipitates of each of these three proteins the remaining two were detected [19]. We demonstrate here that besides RSK1 and PKN also DDX3 was present in YopM-SBP-CBP precipitates (Fig 1A). Interestingly, however, in DDX3-FLAG immunoprecipitates YopM and RSK1 but not PKN was found. Moreover, in PKN-FLAG immunoprecipitates, YopM and RSK1 but not DDX3 was detected (Fig 1E). We conclude from these data that the two partners YopM and RSK can form a triple complex with either DDX3 or PKN but that YopM does not bring PKN and DDX3 together in the same complex. Thus, in addition to the known complex YopM:RSK:PKN, the complex YopM:RSK:DDX3 is formed in cells by the action of YopM.

### A YopM dimer binds to a N-terminal region of DDX3

**Fig 2:**
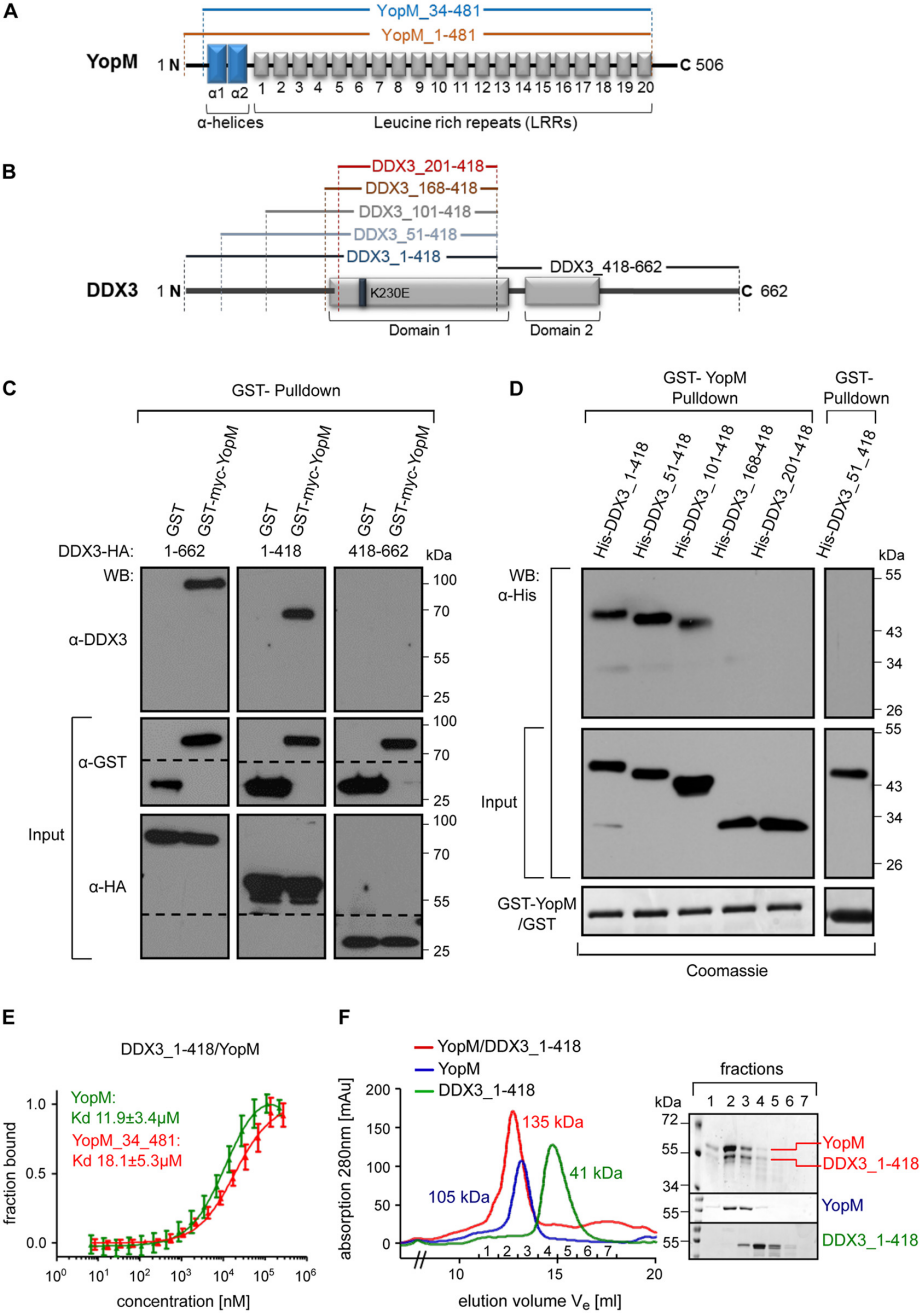
YopM and DDX3 form a 2:1 molecular complex in solution. **A) Schematic representation of YopM from *Y*. *enterocolitica* WA314**. The N-terminal blue rectangles in the YopM scheme correspond to the two α-helices (α1 and α2). LRRs are represented by numbered boxes. YopM_34–481 represents the crystallized variant of YopM (see below) containing all structured features of the protein. **B) Schematic representation of DDX3**. The gray rectangles in the DDX3 scheme correspond to the two helicase core domains (1 and 2). Truncated constructs used in GST-pulldown and immunoprecipitation are marked by amino acid numbers and brackets. **C) GST-YopM pulldown of transfected DDX3 constructs**. GST-Myc-YopM, GST and indicated HA-tagged DDX3 constructs were coexpressed in HEK293T cells and proteins precipitated by glutathione sepharose beads or in input (1/10 volume) were detected by Western blot using indicated antibodies. **D) GST-YopM pulldown of bacterially expressed DDX3 constructs**. Recombinant GST-YopM or GST bound to glutathione sepharose beads were incubated with bacterial lysates containing indicated His-tagged DDX3 constructs. DDX3 proteins precipitated by GST-YopM or in 1/10 lysate input were detected by Western blot using HisProbe-HRP conjugate. Input of GST or GST-YopM is shown in Coomassie stained gels. **E) Microscale thermophoresis determines the binding affinity between YopM and DDX3**. Binding strength of DDX3_1–418 to either YopM or YopM_34–481 was quantified by microscale thermophoresis as in Methods. The dissociation constants (Kd) for the respective interactions are indicated (mean +/- SD; one representative experiment out of three similar ones). For microscale thermophoresis experiment determining the binding strength of YopM to either DDX3_1–418 or DDX3_51–418 see also S1A Fig. **F) Analytical size exclusion chromatography of the YopM:DDX3 complex**. YopM, DDX3_1–418 or a 1:1 (molar ratio) mixture of both proteins were each run on a Superdex 200 gel filtration column. Molecular weights of the peak materials of the three runs (color coded and super-imposed in the figure) were calculated by comparing peak elution fractions with those of standard molecular weight marker proteins (S1B Fig for calibration curves, left panel). Indicated color coded peak fractions were analyzed by SDS PAGE. See also S1C Fig for analytical size exclusion chromatography of the YopM_34–481 and DDX3 1–418 complex. Corresponding calibration curve is shown in S1B Fig (right panel).

To decipher the regions of DDX3 that mediate binding to YopM (Fig 2A) we cotransfected HEK293T cells with vectors expressing GST-myc-YopM and either full length DDX3_1-662-HA, DDX3_1-418-HA or DDX3_418-662-HA, whereby the two truncated DDX3 constructs encompass the recA like domains 1 and -2, respectively (Fig 2B; [31]). GST-pulldowns demonstrated that GST-myc-YopM associated with DDX3_1-662-HA and DDX3_1-418-HA but not with DDX3_418-662-HA (Fig 2C). To test whether the interaction of YopM with DDX3_1–418 is direct and to further narrow down the DDX3 region binding, we performed pulldown experiments using purified GST-YopM and His-fusion proteins of DDX3_1–418 and 4 consecutive N-terminal truncations thereof (Fig 2B). We found that His-DDX3_1–418, His-DDX3_51–418 and His-DDX3_101–418 but not His-DDX3_168–418 or His-DDX3_201–418 bound to GST-YopM (Fig 2D). Thus, the unstructured N-terminus of DDX3 within amino acids 101–168 appears to be required for interaction with YopM.

Microscale thermophoresis (MST) was performed to determine the binding affinity of YopM and DDX3_1–418 or YopM_34–481 and DDX3_1–418 [32]. The YopM_34–481 construct essentially consists of the LRR region and the two N-terminal α-helices that initiate LRR folding (Fig 2A). Dissociation constants (Kd) of 12 μM and 18 μM for the YopM:DDX3_1–418 and the YopM_34–481:DDX3_1–418 interactions, respectively, indicate a medium binding strength and demonstrate that the LRR domain of YopM suffices to bind DDX3 (Fig 2E). Additional experiments showed that compared to DDX3_1–418 the DDX3_51–418 construct retained equal binding affinity to YopM (S1A Fig).

For structural analysis we sought to purify and crystallize a YopM:DDX3 complex. Purified YopM, which has a molecular weight of 55 kDa, eluted at approximately 105 kDa in size exclusion chromatography, which is consistent with a YopM dimer (Fig 2F and S1B Fig). In comparison, purified DDX3_1–418 eluted at around 41 kDa which is indicative of a monomer (Fig 2F and S1B Fig). When YopM and DDX3_1–418 were reacted and subjected to size exclusion chromatography a protein peak containing YopM and DDX3_1–418 eluted at an apparent molecular weight of 135 kDa (Fig 2F and S1B Fig). This molecular weight best fits a 2:1 stoichiometry and supports the notion that the YopM:DDX3_1–418 complex is formed by association of one YopM dimer and one DDX3_1–418 monomer. Similarly, size exclusion chromatography indicated formation of a YopM_34–481:DDX3_1–418 complex running at 127 kDa, again consistent with a 2:1 stoichiometry (S1B and S1C Fig). Finally, size exclusion chromatography also indicated that the YopM isoforms from *Y*. *enterocolitica* 8081 containing 13 LRRs and *Y*. *pseudotuberculosis* YPIII containing 15 LRRs, the latter sharing 99.5% amino acid identity with the isoform from *Y*. *pestis* CO92, also associate with DDX3_51–418 ([14]; S1D Fig).

For crystallization trials we employed a YopM_34–481:DDX3_51–418 complex which proved most stable in solution. However, this complex did not crystallize. We therefore applied small angle X-ray scattering (SAXS) to assess the structure of the YopM_34–481:DDX3_51–418 complex. SAXS allows one to build structural models of macromolecules in solution to a resolution of 10–15 Å [33].

**Fig 3:**
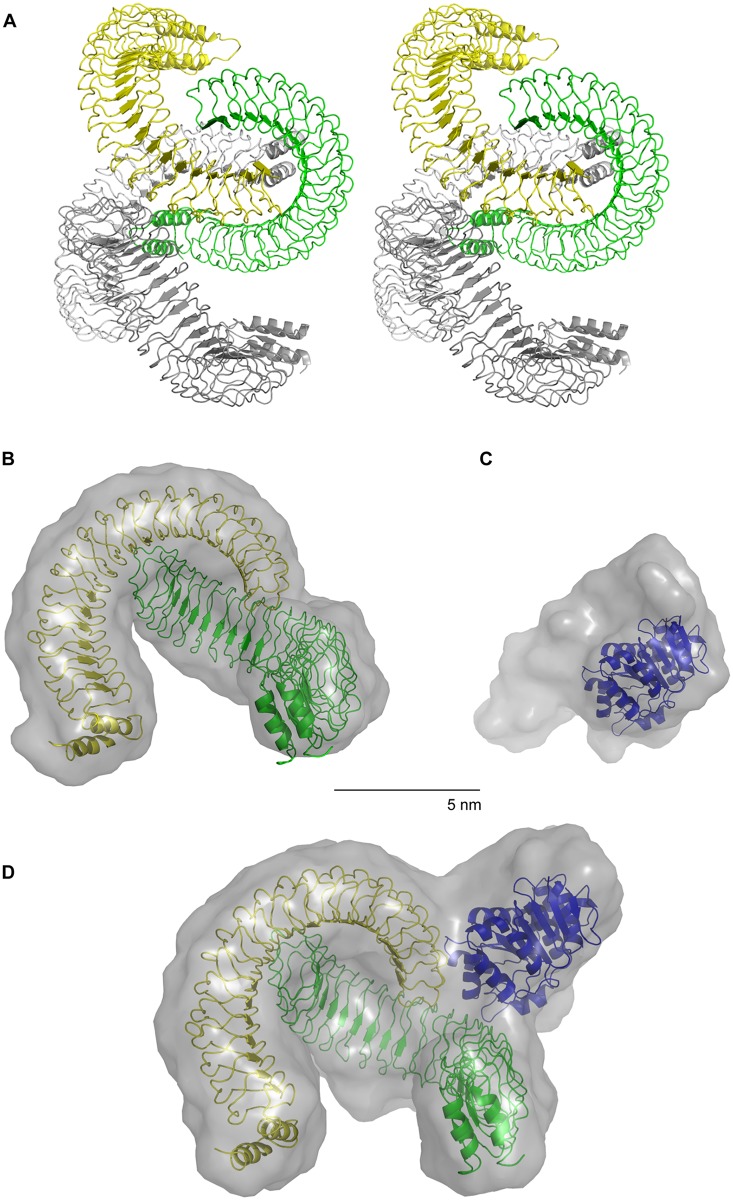
Crystal- and solution structure of YopM_34–481 and solution structures of DDX3_51–418 and the YopM_34-481/DDX3_51–418 complex. **A)** YopM_34–481 was crystallized and its structure was solved (Methods; S2 Table). Stereo view shows ribbon representation of one asymmetric unit of the YopM_34–481 crystal (PDB code 4OW2). The asymmetric unit contains four molecules equivalent to two biological assemblies each represented by a dimer. YopM molecules of one dimer are colored in yellow and green and of the other dimer in light grey and grey. **(B-D)** The YopM_34–481 dimer, the DDX3_51–418 construct and the YopM_34-481/DDX3_51–418 complex were analyzed by small angle X-ray scattering (SAXS). **B)** The SASREF-model (Methods) of YopM_34–481 shown as transparent grey surface representation and the crystal structure of the YopM_34–481 dimer in ribbon representation are superimposed. **C)** The SASREF-model of DDX3_51–418 shown as transparent grey surface representation and the crystal structure of the ATPase domain of DDX3 (residues 167–418, PDB code 2I4I) are superimposed. **D)** The SASREF-model of the YopM_34-481/DDX3_51–418 complex shown as transparent grey surface representation is superimposed with the crystal structures of DDX3 (residues 167–418; [31] and the YopM_34–481 dimer (as presented in A). For experimental SAXS data see S2 Fig and S3 Table.

SAXS data were recorded of the individual YopM and DDX3 constructs and the YopM:DDX3 complex. The computed scattering from the crystal structure of YopM from *Y*. *pestis* 195/P [16] did not agree with the experimental SAXS data of *Y*. *enterocolitica* WA314 YopM_34–481 (yielding a very high discrepancy of χ^2^ = 410). This discrepancy could not be attributed solely to the difference in the number of LRRs (15 LRRs in the crystal structure of *Y*. *pestis* YopM and 20 LRRs in *Y*. *enterocolitica* YopM structure [14]), as the attempts to add the missing LRRs yielded no satisfactory fit. Because this indicated that the overall organization of the two YopM constructs is significantly different we therefore crystallized the *Y*. *enterocolitica* YopM_34–481 construct and solved its structure to 3.2 Å resolution (S2 Table and Fig 3A; PDB code 4OW2).

The crystal structure revealed four molecules in the asymmetric unit (S2 Table and Fig 3A). The asymmetric unit is the smallest part of the crystal structure to which symmetry operations can be applied. Analysis of the interaction interfaces (Methods; [34]) between the four YopM molecules indicated that two YopM molecules mutually interact by binding of the C-terminal LRR 20 (residues 461–481) of one molecule to the internal LRRs 14–17 (residues 342–422) of the other molecule (Fig 3A). The total interface area comprises approximately 1010 Å^2^ and is mediated by 21 hydrogen bridges (S4 Table). The crystallographic dimer fitted the experimental SAXS data reasonably well (χ^2^ = 4.3), which could be further refined to a nearly perfect fit (χ^2^ = 2.3) by using a rigid body modelling to slightly change the angle between the two YopM molecules (Fig 3B and S2 Fig; Methods).

The published crystal structure of DDX3 comprising residues 167–418 [31] was complemented with the N-terminal residues 51–166 (Fig 3C) yielding a very good fit to the SAXS data (χ^2^ = 0.7, S2 Fig, Methods).

The DDX3 model also provides information about the contribution of the N-terminal residues to the overall shape of DDX3_51–418 and confirms our biochemical results suggesting that DDX3 is a monomer in solution (Figs 2F and 3C, S2 Fig).

The SAXS model of the YopM_34–481:DDX3_51–418 complex was finally generated based on the YopM dimer and the SAXS model of DDX3_51–418 (Fig 3D and S2 Fig). This model suggests that the DDX3 molecule (blue colored in Fig 3D) protrudes as a knob-like structure from the complex. It appears that the YopM dimer forms a binding interface for the DDX3 fragment. The first part of the binding interface covers an area of around 1000 Å^2^ and is formed by LRRs 7–9 of the first YopM molecule (green colored in Fig 3D). The second part of the interface covers around 780 Å^2^ and is formed by the C-terminal LRR 20 of the second YopM molecule (yellow colored in Fig 3D). Given the limited resolution, the SAXS data do not allow to narrow down the exact interface region on the side of DDX3 or the position of the N-terminal residues 101–168 essential for YopM binding.

Altogether these data indicate that a dimer represents the smallest biological assembly of YopM and that the dimer creates an interface that allows binding of one molecule of DDX3, thus forming a 2:1 YopM:DDX3 complex.

### DDX3 regulates nuclear export of YopM

**Fig 4:**
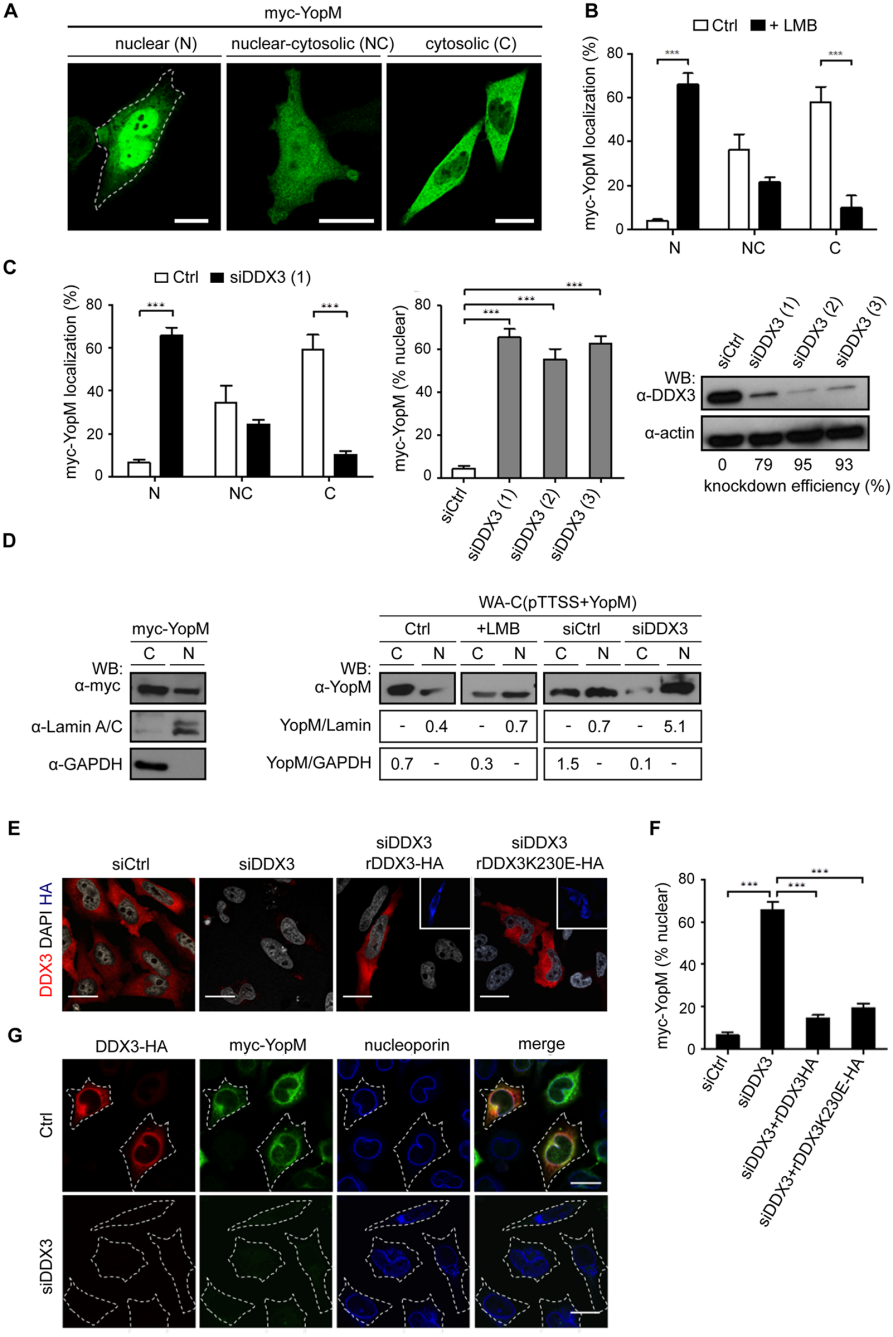
DDX3 mediates nuclear export of YopM via the CRM1 pathway. **A) Defining three patterns of nuclear/cytosolic distribution of YopM**. HeLa cells expressing myc-YopM were immunofluorescence stained using anti-myc antibody and a nuclear (N), nuclear/cytosolic (NC) and cytosolic (C) distribution pattern of YopM was identified. Scale bar, 20μm. **B) CRM1 inhibitor Leptomycin B increases percentage of nuclear localized YopM**. HeLa cells expressing myc-YopM were not treated (Ctrl) or treated with 25 nM LMB for 4 h and then anti-myc immunofluorescence stained. The percentage of cells showing a N-, NC- or C localization of myc-YopM was determined. Each bar represents mean ± SED of 100 cells from three different experiments; ***p<0.001. For distribution of DDX3 within cells under LMB treatment see S3 Fig. **C) Knockdown of DDX3 increases percentage of nuclear localized YopM**. HeLa cells were treated with control siRNA (siCtrl) or DDX3 siRNA (siDDX3 No. 1, 2 or 3 as indicated) for 48 h, transfected with a vector encoding myc-YopM for 18 h and the percentage of cells displaying a N-, NC-, or C localization (left panel) or a N-localization (middle panel) of YopM was determined employing anti-myc immunofluorescence staining. Lysates of siRNA treated cells were analyzed by Western blot using anti-DDX3 antibody to determine knockdown efficiencies. Anti-actin antibody was used to verify equal loading of the SDS PAGE gels (right panel). Each bar represents mean ± SEM of 100 evaluated cells from 2–4 different experiments; ***p<0.001. **D) Inhibition of CRM1 and knockdown of DDX3 increase the amount of YopM in nuclear cell fractions**. (Left panel) Cytosolic (C) and nuclear (N) fractions of HEK293T cells expressing myc-YopM were prepared as in Methods and subjected to anti-myc immunoblot. Purity of fractions was verified using anti-GAPDH (cytosolic marker) and anti-Lamin A/C- antibody (nuclear marker), respectively. (Right panel) HEK293T cells were not treated (Ctrl) or treated with LMB, non targeting siRNA (siCtrl) or DDX3 siRNA (No. 3) and then infected with WA-C(pTTSS+YopM) for 1.5 h. Cytosolic (C) and nuclear (N) fractions of the cells were subjected to anti-YopM, anti-Lamin A/C and anti-GAPDH immunoblot. Band intensity ratios of nuclear YopM/Lamin and cytosolic YopM/GAPDH are depicted. **E) Recomplementation of DDX3 knockdown cells with different DDX3 constructs**. HeLa cells were treated with control siRNA (siCtrl) or DDX3 siRNA (siDDX3 No. 3) for 48 h and then transfected with vectors encoding siRNA-resistent HA-labelled DDX3 (rDDX3-HA) or the K230E mutant thereof (rDDX3K230E-HA). Cells were double immunofluorescence stained with anti-DDX3- and anti-HA antibodies and costained with DAPI for visualizing nuclei. Cells (re)expressing rDDX3 or rDDX3K230E stained positive with both, anti-DDX3- (red) and anti-HA (blue; insets) antibodies. Scale bar, 20 μm. **F) Recomplementation of DDX3 reverses nuclear accumulation of YopM**. HeLa cells were treated as in E) and were additionally transfected with myc-YopM and then immunofluorescence stained with anti-DDX3-, anti-HA and anti-myc antibodies. N-localized YopM was evaluated in cells either negative for DDX3 staining (siDDX3) or positive for DDX3 staining (remaining conditions). Each bar represents mean ± SEM of 100 cells from three different experiments; ***p<0.001. **G) YopM and DDX3 colocalize at the outer nuclear membrane**. HeLa cells were not treated (upper row) or treated with DDX3 siRNA No. 1 for 48 h (lower row) and then transfected with vectors encoding myc-YopM and DDX3-HA. Cells were permeablized with digitonin for 2 min before fixation to release the cytoplasm but leave the outer nuclear membrane intact and then stained with anti-HA- (red), anti-myc- (green) and anti-nucleoporin (blue) antibodies. Representative images are shown. Scale bar, 20 μm.

DDX3 has been implicated in various cellular processes including gene transcription, mRNA-splicing and -export as well as translation. It has also been reported to play a role in cell cycle control, regulation of apoptosis and innate immune signaling [35, 36]. While many of the described DDX3 activities could conceivably be subverted by *Yersinia* YopM, we found it particularly compelling that DDX3 is required for nuclear export of the RNA-binding protein Rev of HIV [37]. DDX3 itself is a nucleocytoplasmic shuttling protein whereby its nuclear export is accomplished by binding to CRM1 [37]. Because Rev was demonstrated to directly interact with DDX3 and inhibition of DDX3 or CRM1 blocked nuclear export of Rev, it was proposed that Rev is transported by binding to the DDX3/CRM1 complex [37]. We reasoned that if DDX3 also mediates nuclear export of YopM, this should be dependent on CRM1 and therefore nuclear levels of YopM should increase by treatment of cells with the CRM1 inhibitor Leptomycin B (LMB). To quantify the cytosolic/nuclear distribution of YopM we first considered that the nuclear levels of YopM may vary considerably between individual cells as reported previously [11]. In fact, when myc-YopM was expressed in HeLa cells and visualized by immunofluorescence, approximately 4% of cells displayed a predominant nuclear localization of myc-YopM (N-localized), in 36% of cells myc-YopM was evenly distributed between cytoplasm and nucleus (NC-localized) and 58% of cells showed a predominant cytoplasmic localization of myc-YopM (C-localized) (Fig 4A and 4B). Upon treatment with LMB the percentage of cells with N-localized myc-YopM increased substantially to 66% whereas the percentage of cells with C-localized myc-YopM decreased to 10% (Fig 4B). LMB also caused a strong nuclear accumulation of DDX3, which confirmed that the reported CRM1-dependent nuclear export of DDX3 also takes place under our experimental conditions (S3 Fig). In order to find out whether DDX3 mediates the CRM1-dependent nuclear export of YopM we knocked down DDX3 using siRNA technology and quantified the cytosolic/nuclear distribution of myc-YopM. Like LMB treatment, DDX3 knockdown induced a strong increase of cells with N-localized myc-YopM and a strong decrease of cells with C-localized myc-YopM (Fig 4C). Three different DDX3 specific siRNAs enhanced nuclear accumulation of myc-YopM to a similar degree (Fig 4C). Fractionation of myc-YopM expressing cells into a cytosolic and a nuclear compartment and anti-myc immunoblotting revealed in accordance with the immunofluorescence data that overall a larger amount of cellular myc-YopM is located in the cytosol than in the nucleus (Fig 4D). In order to demonstrate that also bacterially translocated YopM is exported from the nucleus via CRM1/DDX3 we infected LMB- and siDDX3 treated cells and the respective controls with a *Yersinia* strain translocating YopM (WA-C(pTTSS+YopM)). Cells were fractionated and the YopM levels in the cytoplasmic and nuclear fractions were determined with anti-YopM immunoblot. Also these experiments showed that the YopM amount increased in the nuclear fractions and decreased in the cytosolic fractions upon treatment of the cells with LMB or DDX3 siRNA (Fig 4D).

To investigate whether ATPase and/or helicase activities of DDX3 are required for nuclear export of YopM we cotransfected HeLa cells with DDX3 siRNA and vectors expressing siRNA-resistant, hemagglutinin (HA) wild type DDX3 (rDDX3-HA) or the K230E mutant thereof (rDDX3K230E-HA) (Fig 4E and 4F). The K230E mutation in the Walker A motif of DDX3 causes a loss of both ATPase and helicase activity [37]. Recomplementation of DDX3 depleted cells with rDDX3-HA or rDDX3K230E-HA almost completely reversed the high level of nuclear localized YopM (60–70% of cells) to the low level (10–20%) observed in control cells (Fig 4F).

For a visual corroboration of the DDX3/YopM interaction in the context of nuclear export we took advantage of the finding that after its export from the nucleus DDX3 colocalizes with nucleoporins at the outer side of the nuclear membrane [37]. By extracting cells with digitonin before immunostaining and fixation we could verify that myc-YopM colocalizes with DDX3-HA and nucleoporins at the outer nuclear membrane (Fig 4G). Upon knockdown of DDX3 (endogenous and DDX3-HA) myc-YopM was no longer detectable in the nuclear membrane (Fig 4G).

We conclude that DDX3 independent of its ATPase- and helicase activity mediates nuclear export of YopM via the CRM1 export pathway.

### Nucleocytoplasmic shuttling of YopM controls the phosphorylation status of nuclear RSK1

The question arises which nuclear processes the nuclear localized YopM might regulate. Because YopM increases the phosphorylation of RSK-family kinases and nuclear localized RSK1 has been implicated in transcriptional control [38, 39] we asked whether nuclear YopM might affect the phosphorylation status of RSK1 in the nucleus. RSK1 is phosphorylated on multiple Ser- and Thr-residues through several kinases including its own C-terminal kinase domain [21]. The ERK/MAPK (mitogen-activated protein kinase) cascade directly or indirectly regulates these phosphorylation events [40]. Two of the crucial phosphorylation sites in RSK1 are Ser-380, which provides a docking site for 3'-phosphoinositide-dependent kinase-1 (PDK1) and Ser-221, which is phosphorylated by the docked PDK1 and is thought to activate RSK1 [21].

**Fig 5:**
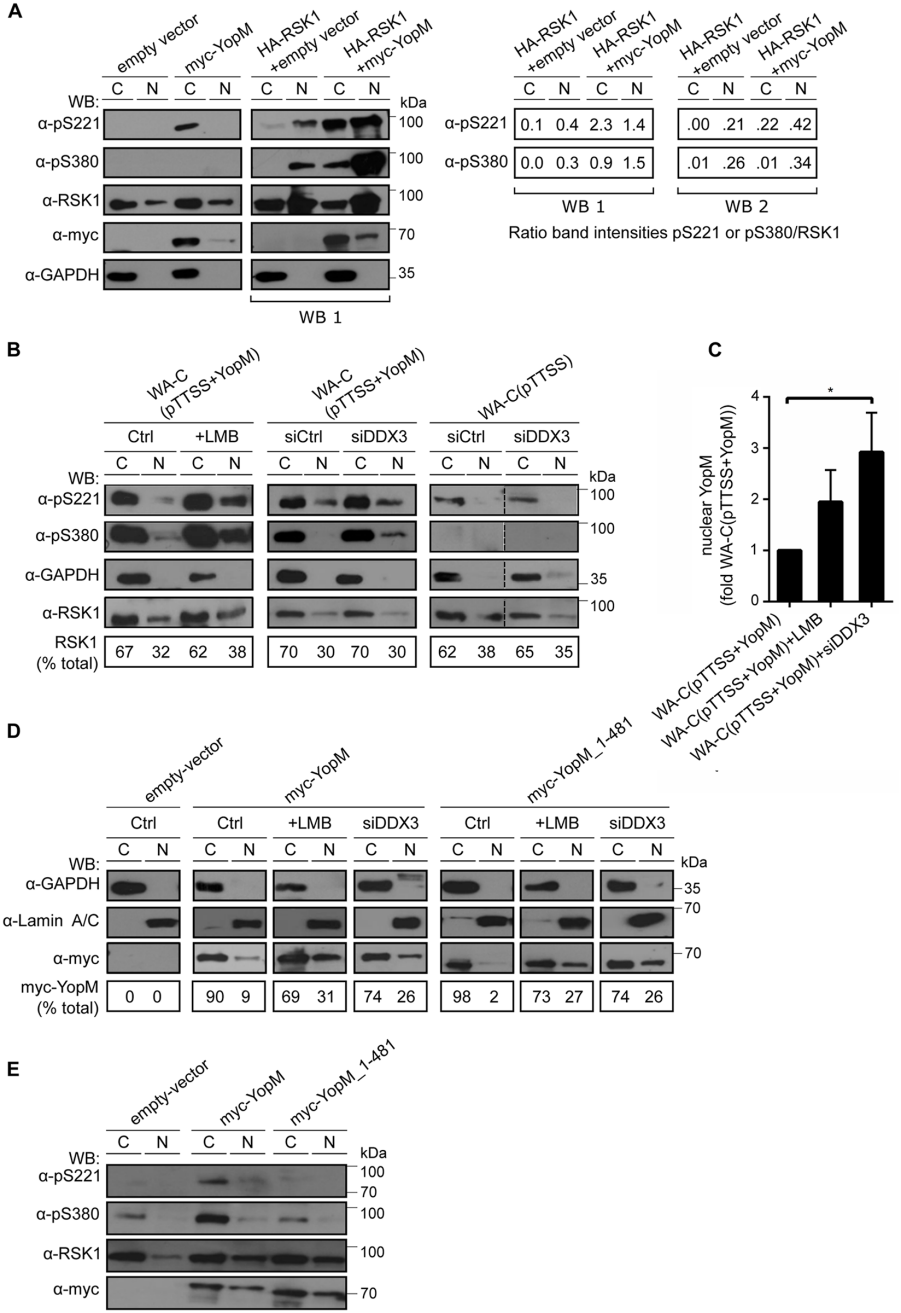
Nuclear YopM controls phosphorylation of nuclear RSK1. **A) Coexpression of HA-RSK1 and myc-YopM increases phosphorylation of nuclear RSK1**. (Left panel) HEK293T cells were transfected with indicated expression vectors and cytosolic- (C) and nuclear (N) fractions were investigated by immunoblot using indicated antibodies. Anti-pS221- and Anti-pS380 antibodies detect the respective phosphorylated amino acids in RSK1. Exposure time of the anti-RSK1 panel in the HA-RSK1 expressing cells (right, WB 1) is about 1/5 of that of the respective panel in control cells (left). (Right panel) Intensities of the pS221- pS380- and RSK1 protein bands from WB 1 and WB 2, the latter derived from a second identical experiment were quantified and intensity ratios of pS221/RSK1 and pS380/RSK1 were calculated. **B) Leptomycin B treatment and DDX3 knockdown increase phosphorylation of nuclear RSK1**. (Left panel) HEK293T cells were not treated (Ctrl) or treated with 25 nM LMB for 4 h. (middle and right panels) HEK293T cells were treated with control siRNA (siCtrl) or DDX3 siRNA (siRNA No. 3) for 24 h. Cells were infected with *Yersinia* WA-C(pTTSS+YopM) (middle panel) or WA-C(pTTSS) (right panel) for 90 min and cytosolic- (C) and nuclear (N) fractions were investigated by immunoblot using indicated antibodies. Bottom panel shows percentage of total RSK1 present in the C- and N fraction of cells in each condition. **C) Leptomycin B treatment and DDX3 knockdown increases nuclear YopM**. HEK293T cells were not treated or treated with 25 nM LMB for 4 h or DDX3 siRNA (No. 3) for 24 h, infected with *Yersinia* WA-C(pTTSS+YopM) for 90 min and nuclear fractions of cells were investigated by anti-YopM immunoblot. Band intensities of YopM signals were determined by ImageJ. Each bar represents mean ± SEM of values from 4 experiments for LMB treatment and 3 experiments for DDX3 siRNA treatment; *p<0.05 (Bonferroni's multiple comparisons test). **D) YopM_1–481 mutant defective in interaction with RSK accumulates in the nucleus and is exported from the nucleus in a LMB- and DDX3 dependent fashion**. HEK293T cells were not treated (Ctrl), treated with 25 nM LMB for 4 h or DDX3 siRNA (siRNA No. 3) for 24 h and transfected with indicated expression vectors. Cytosolic- (C) and nuclear (N) fractions of cells in each condition were investigated by immunoblot using indicated antibodies. Bottom panel shows percentage of total myc-YopM present in the C- and N fraction of cells in the respective condition. See also S4 Fig for immunoprecipitaton of myc-YopM; myc-YopM_1–481 and myc-YopM_34–481 to verify the incapability of YopM lacking the C-terminus to bind RSK. **E) YopM_1–481 mutant is unable to stimulate phosphorylation of RSK**. Indicated myc-YopM constructs or empty vector were expressed in HEK293T cells and cytosolic (C) or nuclear (N) fractions were analyzed by Western blot using indicated antibodies.

Endogenous RSK1 phosphorylated on Ser-221 or Ser-380 could not be detected in the cytosol or nucleus of HEK293T cells expressing empty vector. Upon expression of myc-YopM, RSK1 phosphorylated on Ser-221 became detectable in the cytosol, but not in the nucleus (Fig 5A). RSK1 phosphorylated on Ser-380 could neither be found in the cytosol nor in the nucleus of myc-YopM expressing cells (Fig 5A). In order to increase the cellular level of RSK1 we expressed HA-RSK1 in the HEK293T cells (Fig 5A). Hereupon HA-RSK1/RSK1 phosphorylated on Ser-221 and Ser-380 became detectable in the nuclear fractions of the cells expressing empty myc-vector (Fig 5A, Western blot 1 (WB 1)). Furthermore, upon coexpression of myc-YopM and HA-RSK1 a strong increase in HA-RSK1/RSK1 phosphorylated on Ser-221 and Ser-380 occurred in the cytosolic- as well as in the nuclear cell fractions (Fig 5A, WB 1). Notably, myc-YopM expression did neither alter the total amount nor the cytosol/nucleus distribution of endogenous RSK1 or HA-RSK1/RSK1 (Fig 5A). Thus, by expressing tagged YopM and -RSK1 constructs in cells we can demonstrate that YopM increases phosphorylation of RSK1 in the cytosolic and nuclear cell compartments.

We have previously reported that in cells infected with WA-C(pTTSS+YopM) phosphorylation of endogenous RSK1 on Ser-380 and Ser-221 increases when compared to cells infected with WA-C(pTTSS) [20]. In WA-C(pTTSS+YopM) infected HEK293T cells RSK1 phosphorylated on Ser-380 and Ser-221 could readily be detected in the cytosolic- but barely in the nuclear fractions (Fig 5B). Likely due to a side effect of intensive cell treatment, i.e. cell transfection and -infection in parallel, RSK1 phosphorylated on Ser-221 could regularly be observed in the nuclear fraction of cells that were both infected with WA-C(pTTSS+YopM) and treated with control siRNA (siCtrl; Fig 5B, middle panel). When WA-C(pTTSS+YopM) infected cells were treated with LMB phosphorylation of RSK1 on Ser-380 and Ser-221 became readily detectable in the nuclear fractions (Fig 5B). Similarly, in the nuclear fractions of WA-C(pTTSS+YopM) infected cells pretreated with DDX3 siRNA, phosphorylation of RSK1 on Ser-380 or Ser-221 became detectable or slightly increased, respectively (Fig 5B). As observed already in myc-YopM expressing cells (Fig 5A), the level of total cellular RSK1 and its distribution between nucleus and cytosol were not altered by bacterially injected YopM and were also unaffected by LMB- or DDX3 siRNA pretreatment (Fig 5B). LMB treatment tended to increase and DDX3 knockdown significantly increased the nuclear level of YopM (Fig 5C). Infection with WA-C(pTTSS) in combination with LMB- or DDX3 siRNA treatment did not increase nuclear RSK1 phosphorylation (Fig 5B). Taken together, these results suggest that phosphorylation of nuclear RSK1 correlates with the nuclear level of YopM and is not due to enhanced translocation of RSK1 to the nucleus. To find out whether a direct interaction of YopM and RSK1 is required for the increased nuclear RSK1 phosphorylation we employed myc-YopM_1-481- and myc-YopM_34–481 expression constructs, which lack the C-terminal amino acids required for RSK binding [7]. RSK1 did neither coimmunoprecipitate with myc-YopM_1–481, which confirms earlier work [7] nor with myc-YopM_34–481. DDX3 and PKN coimmunoprecipitated with both of these constructs (S4 Fig). In response to LMB- or siDDX3 treatment the RSK1-binding deficient myc-YopM_1–481 accumulated in the nucleus of cells to a similar degree as control myc-YopM (Fig 5D). As expected, myc-YopM_1–481 did not increase phosphorylation of RSK1 (Fig 5E). Thus, YopM requires a direct interaction with RSK for increasing RSK phosphorylation in the cytosol and nucleus of cells but nuclear import and export of YopM appears to be unaffected by interaction with RSK.

In summary, we conclude that nucleocytoplasmic shuttling of YopM adjusts the phosphorylation level but not the amount of RSK1 in the nucleus.

### YopM induces transcription of IL-10 in primary human macrophages

In infected animals the eminent role of YopM for virulence of *Yersinia* has been experimentally related to the subversion of cytokine gene expression and -production [8, 25]. However, two independent studies using whole genome microarrays were unable to detect an effect of YopM on transcription of immune mediator genes in macrophages [41, 42].

Because YopM affects phosphorylation of RSK, an established regulator of gene transcription, and recent studies showed inhibition or stimulation of individual cytokine gene expression by YopM in macrophages [4, 10], we reinvestigated the impact of YopM on the global transcriptional response of *Yersinia* infected macrophages. To this end total RNA was prepared from duplicate samples of primary human macrophages infected for 1.5 h or 6 h without (mock group) or with *Yersinia enterocolitica* WA314 (WA314 group) or YopM deletion mutant WA314 (WA314ΔYopM group) and subjected to RNA-seq analysis.

When comparing WA314- and WA314ΔYopM infected macrophages at the 1.5 h time point, only 6 genes registered as being differentially expressed (differentially expressed genes, DEGs; log2-fold change ≥ 1; adjusted p-value ≤ 0.05) (S5 Table). Notably, these genes which are all downregulated in the WA314ΔYopM group compared to the WA314 group and therefore are upregulated by YopM include IL-10. WebGestalt analysis revealed that 2 Kyoto Encyclopedia of Genes and Genomes (KEGG) pathways are significantly enriched in these DEGs (adjusted p value ≤ 0.05; S6 Table): i) Cytokine-cytokine receptor interaction and ii) Jak-STAT signaling pathway.

**Fig 6:**
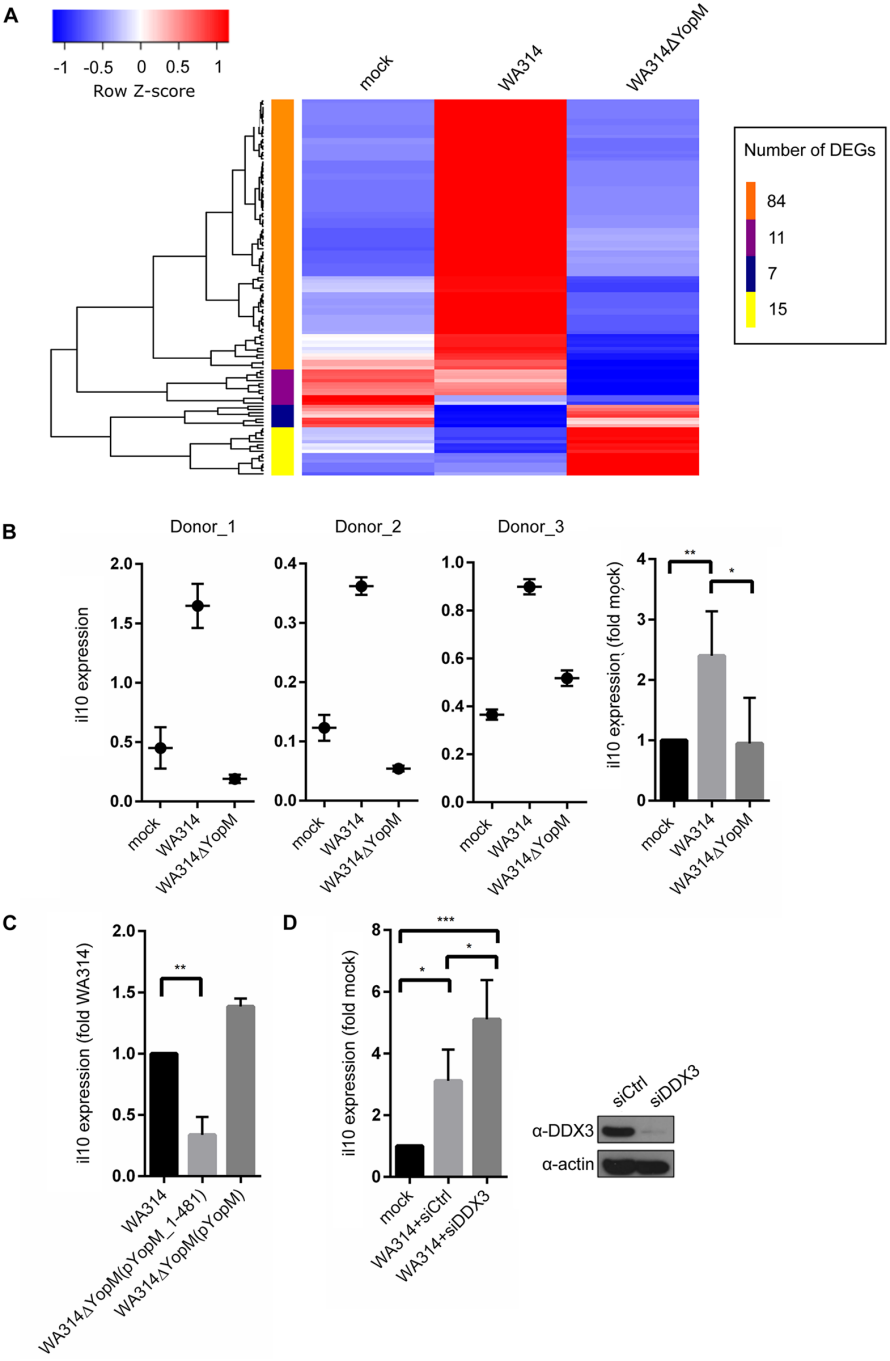
YopM upregulates IL-10 expression in *Yersinia* infected human macrophages. **A) Heatmap of expression values of differentially expressed genes (DEGs) in human macrophages not infected (mock) or infected with WA314 or WA314ΔYopM**. Duplicates (two different donors) of primary human macrophages were infected without (mock) or with *Yersinia enterocolitica* WA314 or WA314ΔYopM for 6 h. Total RNA was prepared from each sample and subjected to RNA-seq. The scaled expression of each set of replicates, denoted as the row Z-score, is plotted in a red-blue color scale. Red indicates high and blue indicates low expression. Only genes with an absolute log2-fold change greater or equal 2 and p-value smaller or equal 0.01 are shown. These genes were hierarchically clustered (complete linkage) according to their expression profiles. The resulting distinct sets of genes are indicated by the orange-, purple-, blue- and yellow color bar. The number of DEGs in each cluster is indicated. **B) Analysis of IL-10 expression in *Yersinia* infected human macrophages from different donors**. Total RNA was isolated from primary human macrophages that were mock infected or infected with WA314 or WA314ΔYopM for 6 h. The RNA was subjected to quantitative RT-PCR using human IL-10 specific primers. IL-10 expression was normalized to expression of three housekeeping genes (GAPDH, TBP, B2M). For each condition triplicate samples of macrophages derived from seven different donors (Donor_1 to Donor_7) were investigated (data from Donor_4 to Donor_7 in S5 Fig). Each bar in graph represents mean ± SD of values from all 7 donors; **p<0.01, *p<0.05. **C) Induction of IL-10 expression requires interaction of YopM with RSK**. Experimental procedures as in B) with the difference that macrophages were infected with WA314ΔYopM(pYopM_1–481) and WA314ΔYopM(pYopM). Each Bar in graph represents mean ± SD of values from 3 different donors; **p<0.01. **D) DDX3 knockdown increases IL-10 expression in *Yersinia* infected human macrophages**. Primary human macrophages were transfected with control siRNA (mock, siCtrl) or DDX3 siRNA (siDDX3 No. 3) for 72 h and not infected (mock) or infected with WA314 for 6 h. Total RNA was subjected to quantitative RT-PCR as in B). Each Bar in graph represents mean ± SD of values from 4 different donors; *p<0.05, ***p<0.001. Western blot verifies DDX3 knockdown in the macrophages.

Major differences in gene expression profiles between the WA314- and WA314ΔYopM groups were identified after 6 h of infection. In total 147 DEGs (22 up- and 125 downregulated) were identified between the WA314ΔYopM- and the WA314 group (http://www.ebi.ac.uk/ena/data/view/PRJEB10086). A heatmap showing expression values of all DEGs in the mock-, WA314- and WA314ΔYopM groups is presented in Fig 6 (log2-fold change ≥ 2; adjusted p-value ≤ 0.01; Fig 6A). Genes were clustered in four main sets marked by orange-, purple-, blue- or yellow color that contain 84, 11, 7 or 15 genes, respectively (Fig 6A).

The orange cluster comprises genes that are upregulated in the WA314 group compared to the mock group and downregulated in the WA314ΔYopM group compared to the WA314 group (Fig 6A). The expression levels of these genes are similar in the mock- and WA314ΔYopM groups. Thus, these genes are upregulated by YopM during infection. They include IL-10 found already to be upregulated by YopM at 1.5 h post infection. WebGestalt analysis revealed that 18 KEGG pathways are significantly enriched in this cluster (adjusted p value ≤ 0.01; S7 Table). Prominent pathways upregulated by YopM include the Jak-STAT signaling pathway, Toll-like receptor signaling pathway and Cytokine-cytokine receptor interaction.

The purple cluster mostly contains genes that are downregulated in the WA314ΔYopM group compared to both, the mock- and the WA314 group. The blue cluster contains genes that are upregulated in the WA314ΔYopM group compared to the WA314 group but unchanged compared to the mock group. The yellow cluster mostly comprise genes that are upregulated in the WA314ΔYopM group compared to both the mock- and the WA314 groups (Fig 6A). As opposed to the orange cluster, no KEGG pathways were significantly enriched in the purple-, blue- or yellow clusters (http://www.ebi.ac.uk/ena/data/view/PRJEB10086).

**Fig 7:**
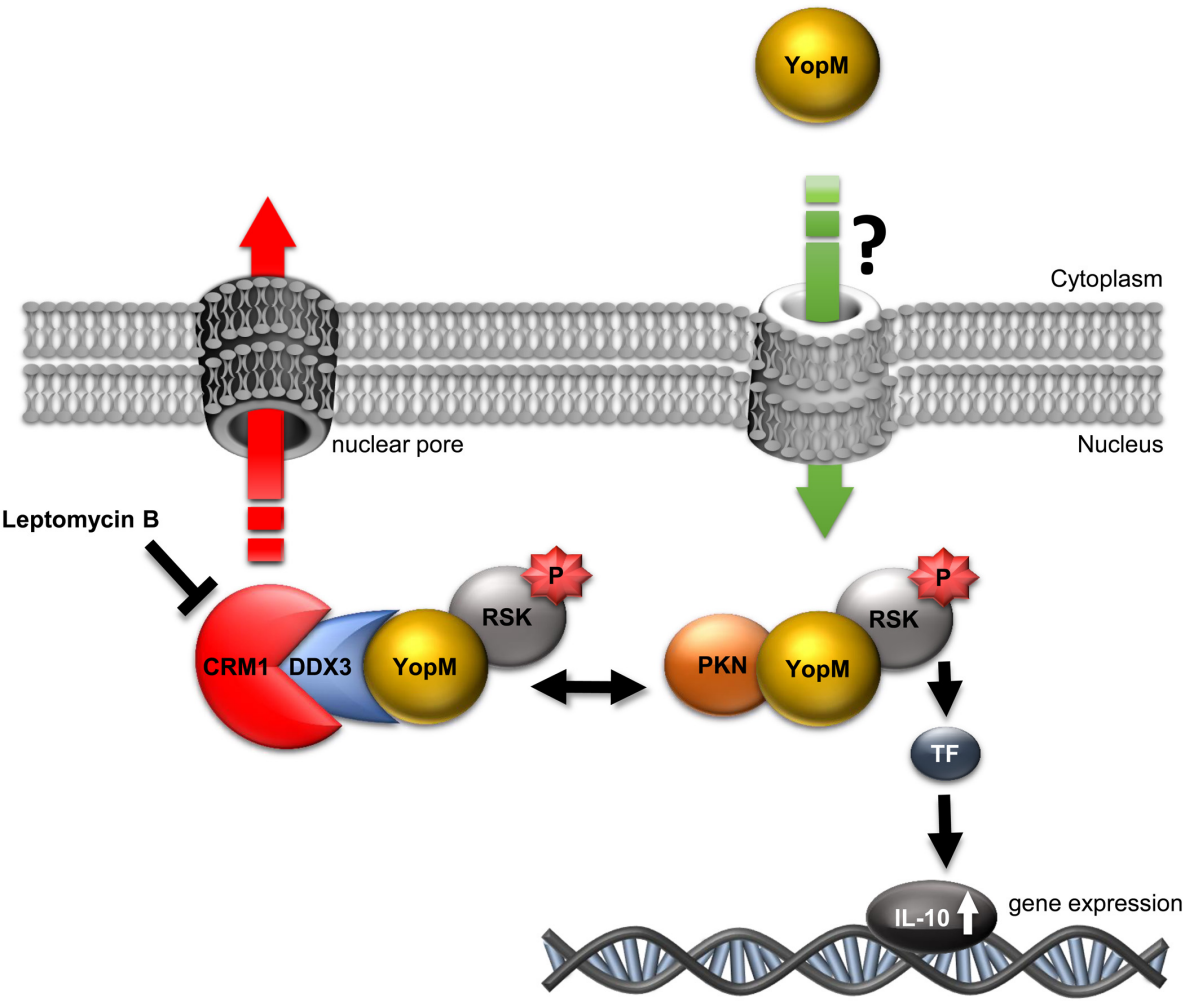
Model of nuclear shuttling and intranuclear activity of YopM. YopM enters the nucleus from the cytoplasm of infected cells through an unknown pathway. In the nucleus YopM forms at least two complexes: YopM:RSK:PKN and YopM:RSK:DDX3. Hyperphosphorylated RSK within the complex induces IL-10 expression. DDX3 mediates nuclear export of YopM via the LMB-sensitive CRM1 export pathway. Abbreviations: RSK, Ribosomal S6 kinase; PKN, Protein kinase N; DDX3, DEAD box helicase 3; CRM1, chromosome region maintenance 1; P, phosphate group; TF, transcription factor.

Because according to RNA-seq analysis IL-10 mRNA was upregulated by YopM already after 1.5 h and also after 6 h of infection (orange cluster) and IL-10 protein levels were previously found to be elevated by YopM in the serum of infected mice [8, 29], we sought to confirm and further analyze the YopM effect on IL-10 expression. For this, macrophages from 7 different human donors were not infected (mock) or infected with WA314 or WA314ΔYopM for 6 h and investigated by quantitative real time PCR (Fig 6B and S5 Fig). Although IL-10 expression in the macrophages from the different donors varied by one order of magnitude, in all cases IL-10 expression levels were increased in the WA314 infected compared to the mock- or WA314ΔYopM infected cells (Fig 6B and S5 Fig). To test whether direct binding of YopM to RSK1 is required for the induction of IL-10 expression we infected macrophages with WA314ΔYopM complemented with YopM_1–481 (WA314ΔYopM(pYopM_1–481); Table 1) or with native YopM (WA314ΔYopM(pYopM)). When compared to IL-10 expression in WA314- and WA314ΔYopM(pYopM) infected macrophages, IL-10 expression was significantly lower in macrophages infected with strain WA314ΔYopM(pYopM_1–481) translocating a YopM mutant that interacts with DDX3 and PKN/PRK but not with RSK1 (S4 Fig, Fig 6C). These data suggest that YopM requires a direct interaction with RSK to induce IL-10 expression in macrophages. Finally, the effect of DDX3 knockdown, which increases YopM level and RSK1 phosphorylation in the nucleus (Figs 4D, 5B and 5C), on IL-10 expression was investigated. DDX3 knockdown further increased IL-10 expression in WA314 infected macrophages (Fig 6D). Altogether these results are consistent with the notion that the nuclear level of YopM is controlled by DDX3-mediated nuclear export of YopM through the CRM1 pathway. Nuclear YopM stimulates nuclear RSK1 phosphorylation and thereby upregulates IL-10 expression (Fig 7).

**Table 1:** *Yersinia* strains used in this study.

Strains or plasmids	Relevant characteristic/Description	Reference of source
WA314	*Y*. *enterocolitica* serotype O:8; clinical isolate; pYVO8+	[43]
WA314ΔYopM(pYopM-SBP-CBP)	WA314ΔYopM strain complemented with the DNA construct YopM-SBP-CBP in pACYC184	[20]
WA314ΔYopM(pYopM)	WA314ΔYopM complemented with YopM in pACYC184	[6]
WA314ΔYopE(pYopE-SBP-CBP)	WA314ΔYopE strain complemented with the DNA construct YopE-SBP-CBP in pACYC184	this study
WA-C(pTTSS)	Virulence plasmid cured *Yersinia enterocolitica* strain WA-C harboring the plasmid pTTSS encoding the TTSS secretion/translocation apparatus of WA314 but no Yop effector genes	[43, 44]
WA-C(pTTSS+YopM)	WA-C(pTTSS) complemented with YopM in pACYC184	[44]
WA314ΔYopM(pYopM_1–481)	WA314ΔYopM complemented with YopM_1–481 in pACYC184	this study

## Discussion

YopM is one of the most important virulence factors of pathogenic yersiniae in mouse models of yersiniosis [6]. Upon injection into host cells by the bacterial type three secretion system YopM displays a cell dependent, variable distribution between cytosol and nucleus [11]. Here we provide evidence that DDX3 mediates nuclear export of YopM via the CRM1 export pathway and propose that this export mechanism together with the entry process, whose molecular basis is unknown, enables nucleocytoplasmic shuttling of YopM. Because the nuclear level of YopM increased when its nuclear export was blocked, we conclude that nucleocytoplasmic shuttling adjusts the level of YopM in the nucleus. The nuclear level of YopM in turn determined the degree of phosphorylation of nuclear RSK1. Using *Y*. *enterocolitica* infected primary human macrophages we were able to show that YopM through its interaction with RSK is responsible for an increased expression of the immunosuppressive cytokine IL-10.

DDX3 is a member of the DEAD/H box family of RNA helicases that have been implicated in all essential steps of gene expression in eukaryotes including transcription, mRNA processing and -export as well as translation [45, 46]. It is expressed in a wide range of tissues and cell lines as well as in primary human macrophages (our own results, Fig 6D), lymphocytes, dendritic cells and natural killer (NK) cells which are the cell types known to interact with *Yersinia* [47–51]. DDX3 has been linked to numerous cellular activities such as cell cycle control, regulation of apoptosis and innate immune signaling [35, 36]. It has also been described to shuttle between the cytosol and the nucleus with its export from the nucleus being mediated by CRM1 [37]. Although to our knowlegde this is the first report implicating DDX3 as a direct bacterial target, DDX3 has been shown before to mediate nuclear export of the RNA-binding protein Rev of Human Immunodeficiency Virus [37]. Furthermore, Hepatitis C virus, Hepatitis B virus and poxviruses target DDX3 and either require it for their replication or subvert its immune regulatory functions [35, 48]. Thus, with our work DDX3 emerges as a central eukaryotic target not only for pathogenic viruses but also for bacteria. Whether, in addition to nuclear export, YopM hijacks or subverts other DDX3 functions such as those described above deserves further investigation.

Numerous interaction partners of the different YopM isoforms have been described, and include the extracellular proteases thrombin and trypsin, kinases of the RSK- and PKN/PRK families, the scaffolding protein IQGAP1 (IQ motif-containing GTPase-activating protein 1) and recently also Caspase-1 [27, 52–55]. Although not many of these interactions have been reconstituted with purified proteins in vitro, the available data indicate that most interaction partners bind to the LRR region of YopM. An exception is RSK1, which binds to the unstructured C-terminal tail of YopM [7]. A YLTD motif in the 10th LRR of two YopM isoforms (from *Y*. *pseudotuberculosis* YPIII and *Y*. *pestis* CO92) was proposed to act as a pseudosubstrate caspase-1 inhibitor [27]. Caspase-1 is required for maturation and secretion of IL-1β in the context of inflammasome activity [56]. However, other work demonstrated that the YLTD motif was dispensable for caspase-1 inhibition and instead a part of the LRR region and the C-terminal tail of YopM were involved in inhibiting caspase-1 [28]. In the latter work IQGAP1 was suggested to mediate the inhibition of caspase-1 by YopM from *Y*. *pestis* KIM but not by YopM32777 from *Y*. *pseudotuberculosis* [28].

Our biochemical and SAXS data indicate that YopM forms a dimer in solution that associates with one molecule of DDX3. Although the SAXS data provide rather low resolution information about the YopM:DDX3 binding interface they suggest that the dimer creates a interaction surface for DDX3. It will require a high resolution crystal structure of the YopM:DDX3 complex and YopM mutants defective in dimerization to further test this notion. YopM's highly conserved unstructured C-terminal tail is not part of the previously published crystal structure [16] or the novel crystal structure obtained in this work. Yet, our structural data suggest that the C-terminal tails of the two YopM molecules that bind DDX3 are still accessible for RSK1. Consistent with this we detected a ternary complex consisting of YopM:RSK1:DDX3 and confirmed the existence of the ternary complex YopM:RSK1:PKN under our conditions [19]. At the same time we could not observe a quadruple complex containing YopM, RSK1, PKN and DDX3. Given that DDX3 and PKN both bind to the LRR region of YopM (this work and [8]) they might compete for this region and thus exclude each other from the same YopM complex. Further studies are necessary to clarify whether the YopM dimer can in fact bind only one or potentially more interaction partners via its LRR region and how these associations are controlled. Because in the YopM:PKN:RSK complex RSK was proposed to phosphorylate PKN [19], phosphorylation by RSK may govern the interaction of proteins with the LRR region of YopM.

DDX3 consists of a helicase core highly conserved in DEAD box helicases [57, 58] that commences approximately at amino acid residue 150 and ends at around residue 550 (Fig 2A). The helicase core in DEAD box proteins is typically flanked at its N- and C-terminus by variable sequences that function in helicase targeting and regulation [31]. In DDX3 the C-terminal 260 to 517 residues associate with the exportin CRM1 [37] and we found that residues 101 to 168 are involved in YopM binding. Thus, when DDX3 associates with a YopM dimer through its N-terminus, its C-terminus would be accessible for concomitant binding of CRM1. We envisage that DDX3 forms a bridge between YopM and CRM1 and thereby promotes YopM's nuclear export. That helicase and ATPase activities of DDX3 are not required for mediating export of YopM is consistent with this notion. We cannot, however, exclude the formal possibility that DDX3 and YopM bind independently to CRM1.

We found that the extent of RSK1 phosphorylation in the nucleus correlated with the nuclear level of YopM. The current concept is that growth-, proliferation- and inflammation promoting stimuli cause activation of ERK 1/2 kinases which phosphorylate RSK [59–61]. Fully phosphorylated and active RSK1 translocates to the nucleus where it is thought to control a plethora of substrates [39, 62]. Specifically, phosphorylation of transcription factors in the nucleus has been proposed to underly the effects of RSK1 on gene expression, cell motility and protein synthesis [63–66]. At present we do not know through which downstream mechanisms the artificially hyperactivated RSK affects gene transcription in the nucleus.

Although in mouse models of yersiniosis an immunosuppressive activity was attributed to YopM and the expression of individual cytokines was modulated by YopM in monocytic cells/macrophages [8, 10, 25, 26], a systematic effect of YopM on the transcriptional immune response has not been described. In the contrary, two earlier transcriptome analyses using gene microarrays did not detect a YopM effect at all [41] or merely an effect on transcription of genes involved in cell cycle- and growth control [42]. In those studies the murine macrophage lines J774A.1 or PU5–1.8 were infected with suitable *Yersinia* strains for 1 h or 2.5 h, respectively [41, 42]. We essentially confirm this previous work by demonstrating that only 6 genes were differentially expressed in human macrophages infected with a YopM deficient- compared to a wild type *Yersinia* strain for 1.5 h. However, it was interesting to note that all of these genes were upregulated by YopM and included IL-10. Furthermore, when macrophages were infected for 6 h a significant YopM-dependent upregulation of > 80 genes was discovered. The 2-3-fold upregulation of IL-10 mRNA at this time point fits well to the 4-fold increase of IL-10 protein induced by YopM in infected mice [8, 29]. Thus, in apparent contradiction to the general virulence strategy of *Yersinia* that involves widespread suppression of immune response genes through the MAP-kinase- and NFκ-B inhibitor YopP/YopJ, YopM upregulates expression of a subset of these genes [41, 42]. Although the YopM effect on macrophage gene expression after 6 h of infection may be due to complex autocrine mechanisms and feed back loops, immunosuppressive mediators like IL-10 whose expression was upregulated after 1.5 h of infection already could drive this response. The positioning of the RSK-family of kinases in the nucleus and their well accepted role in phosphorylating transcription factors could qualify the RSKs as YopM targets responsible for stimulation of transcription. Consistent with this, the induction of IL-10 expression depended on the RSK binding region of YopM. However, more direct proof for this notion would require to shut down all four RSK isoforms at once. That RSKs are principally involved in the immunosuppressive action of YopM was also documented by a recent study showing that *Y*. *pseudotuberculosis* mutants expressing YopM proteins unable to interact with RSK1 are strongly reduced in virulence and suppression of IL-1β secretion by macrophages [28].

YopM has multiple interaction partners in cells and here we provide evidence that by interaction with DDX3 its nuclear level is controlled. Within the nucleus YopM binds and controls phosphorylation of RSK, a downstream effector of the ERK signal pathway. By employing RNA-seq technology and human macrophages we were able to demonstrate that YopM upregulates a number of genes belonging to the immune response, with upregulation of the prominent immunosuppressive cytokine IL-10 already after 1.5 h post infection. It remains a task for future work to decipher exactly which interaction partners and intranuclear activities contribute to the fascinating network of YopM's immunosuppressive activities.

## Materials and Methods

### Bacterial strains

*Yersinia enterocolitica* strains used in this study are derivatives of the serotype O:8 strain WA314 harboring the virulence plasmid pYVO8 or of a plasmid cured version of WA314 named WA-C [43]. WA314ΔYopM was constructed by replacing the YopM gene in WA314 with a kanamycin resistance cassette [6]. WA314ΔYopM(pYopM), WA314ΔYopM (pYopM_1–481) and WA314ΔYopM(pYopM-SBP-CBP) strains were created by complementation of WA314ΔYopM with isogenic full length YopM, YopM_1–481 or YopM-SBP-CBP in vector pACYC184, respectively [20]. WA314ΔYopM(pYopM) and WA314ΔYopM(pYopM_1–481) secreted similar amounts of YopM upon Ca^++^ depletion [43, 44]. WA-C(pTTSS) and WA-C(pTTSS+YopM) harbor the pTTSS plasmid encoding the TTSS secretion/translocation apparatus of WA314 alone or in combination with YopM integrated in pACYC184 [44]. WA314ΔYopE was constructed by replacing the YopE gene with a kanamycin resistance cassette [6] and WA314ΔYopE(pYopE-SBP-CBP) was created by complementation of WA314ΔYopE with an *Y*. *enterocolitica* serotype O9 strain E40 YopE-SBP-CBP construct integrated in pACYC184 [67]. Table 1 provides an overview of the bacterial strains used in this study.

### Expression plasmids

The following expression plasmids have been described previously: HA-tagged RSK1 (Addgene plasmid 13841) [62] and FLAG-tagged PKN1 [68]. For bacterial protein expression the gene for full length YopM (amino acids 1–506) or YopM_ 34–481 was amplified via PCR from virulence plasmid pYVO8 and cloned via restriction/ligation into pGEX-4T2- (GE-Healthcare, Munich, Germany) or pET302 (Thermo Fisher Scientific, Waltham, USA) vectors in which a recognition site for tobacco etch virus protease (TEV) was introduced using suitable primers (S8 Table). Genes of YopM isoforms from *Y*. *enterocolitica* 8081 (amino acids 1–366) and *Y*. *pseudotuberculosis* YPIII (amino acids 1–409) were amplified via PCR from the respected virulence plasmids, generous gifts of Jürgen Heesemann, and cloned into pET302 (Thermo Fisher Scientific, Waltham, USA). For expression in eukaryotic cells YopM constructs were amplified via PCR from virulence plasmid pYVa127/90 (accession NC_004564.1) and cloned into pCS2+MT (XB-VEC-12442480) via the restriction sites NcoI and XbaI resulting in N-terminally myc-tagged YopM (myc-YopM). For the expression of GST-YopM, YopM was derived from the myc-YopM construct via restriction sites BamHI and NotI and ligated into the pEBG-2T vector [69]. His-DDX3 constructs were amplified from human cDNA (accession NM_001356) and cloned via restriction/ligation into the bacterial expression vector pET303 resulting in C-terminal tagged proteins. For expression in eukaryotic cells DDX3 was also amplified from human cDNA (accession NM_001356) and subsequently cloned into the pCDNA3.1 + vector (Invitrogen/Life Technologies, Thermo Fisher Scientific, Waltham, USA) using the restriction sites Hind III and BamHI resulting in the constructs DDX3-HA, DDX3_1-418-HA and DDX3_418-662-HA. HA-tags where introduced by employing suitable reverse primers (S8 Table). The ATPase/helicase deficient DDX3 (DDX3-K230E) [37] and the siRNA No. 3 resistant DDX3 mutant (rDDX3) were constructed using the QuickChange II Site-directed Mutagenesis Kit (Agilent technologies, California, USA) following the manufacturer’s instruction. An overview of the plasmids designed in this study is given in S8 Table.

### RNAi

DDX3X (Gene ID: 1654) siRNA No. 1 (siRNA-ID: s4004, target sequence GCUGAUCGGAUGUUGGAUtt) was from Life Technologies (Carlsbad, USA), siRNAs No. 2 (D-006874-01, target sequence GCAAAUACUUGGUGUUAGA) and 3 (D-006874-02, target sequence ACAUUGAGCUUACUCGUUA) were from Thermo Scientific (Waltham, Massachusetts) and control siRNA (firefly luciferase) was from the Dharmacon siRNA collection (Lafayette, USA).

### Antibodies

Rabbit anti-YopE and -anti-YopM polyclonal antiserum was a generous gift of Jürgen Heesemann (LMU, München, Germany) [70, 71]. Rat anti-YopM monoclonal antibody (9552) was produced by employing the YopM gene from *Y*. *enterocolitica* WA314 and derived YopM recombinant protein as specified recently [72].

Commercially available antibodies used in this study were: (Diluted 1:1000) Monoclonal mouse anti-DDX3 antibody (Santa Cruz Biotechnology), polyclonal rabbit anti-DDX3 antibody (Bethyl Laboratories), monoclonal anti-RSK1(C-21) antibody (Santa Cruz Biotechnology), polyclonal goat anti-PKN1 (C-19) antibody (Santa Cruz Biotechnology), monoclonal rabbit anti-GST antibody (Invitrogen), monoclonal mouse anti-GST (Novus Biologicals, Littleton, USA), monoclonal mouse anti-MAb414 (Covance, Princeton, USA), monoclonal rabbit Lamin A/C (Cell Signaling), monoclonal mouse anti-FLAG (Sigma-Aldrich), monoclonal mouse anti-myc 9B11 antibody (Cell Signaling), polyclonal rabbit myc-tag antibody (Cell Signaling), anti-HA high affinity (Roche), monoclonal rat anti-HA antibody (Santa Cruz Biotechnology), anti-phospho-S380RSK (Cell Signaling); (Diluted 1:2000) anti-phospho-S221RSK (R&D systems), polyclonal anti-GAPDH (Sigma-Aldrich), monoclonal anti-actin (Millipore, Schwalbach, Germany); (Diluted 1:2500) HisProbe-HRP conjugate (Thermo Scientific, Rockford, USA). Secondary antibodies for immunofluorescence were AlexaFluor-488- or AlexaFluor-568-labeled goat anti-mouse, -anti-rabbit IgG and -anti-rat IgG (Molecular Probes, Karlsruhe, Germany). Secondary antibodies for Western blot were horseradish peroxidase linked sheep anti-mouse IgG (GE Healthcare), donkey anti-rabbit IgG F(ab´)2 (Amersham Biosiences), -anti-goat IgG (Santa Cruz Biotechnology) and -anti-rabbit IgG (Cell signaling) and goat anti-rat IgG (GE Healthcare). All used in a dilution of 1:10000.

### Cell culture

HeLa cells (ACC# 57, DSMZ-German Collection of Microorganisms and Cell Cultures) were cultured in DMEM-GlutaMAX-I (Invitrogen, GIBCO Darmstadt, Germany) supplemented with 10% FCS and 5% non-essential amino acids (Sigma-Aldrich, Steinheim, Germany).

HEK293T cells (ATCC# CRL-11268) were grown in DMEM with 10% FCS containing 100 IU/ml Penicillin and 100 μg/ml Streptomycin (all from GIBCO).

J774A.1 (ATCC# TIB-67) cells were grown in RPMI1640 (Invitrogen, GIBCO Darmstadt, Germany) supplemented with 10% FCS, 100 IU/ml Penicillin and 100 μg/ml Streptomycin and additional 2 mM Glutamine. All cell lines were cultured in a humidified 5% CO2 atmosphere at 37C and passaged every 48 hours.

Human peripheral blood monocytes were isolated from buffy coats (provided by Frank Bentzien, University Medical Center Eppendorf, Hamburg, Germany) as described previously [73]. Approval for the analysis of anonymized blood donations (WF-015/12) was obtained by the Ethical Committee of the Ärztekammer Hamburg (Germany). Cells were cultured in RPMI1640 containing 20% autologous serum with medium changes every three days and used for *Yersinia* infection after 2 weeks.

### Transfection of cells

HeLa cells were transfected with Lipofectamine LTX (Life Technologies, Carlsbad, California) according to the manufacturer’s instruction. HEK293T cell were transfected with the calcium phosphate method [74] or with polyethylenimine (PEI) (Polyscience Inc, Pennsylvania, USA) according to the manufacturer’s instruction. RNAi transfection was carried out with Lipofectamine RNAiMAX transfection reagent (Life Technologies, Carlsbad, California) according to the manufacturer’s instruction. siRNA transfection was either performed 24 h after transfection of the cells with the calcium phosphate method or 16–24 h before transfection of the cells with PEI-or Lipofectamine LTX transfection reagent. Cells were usually analyzed 24–48 h after the last transfection. siRNA transfection of human primary macrophages were performed using the Neon Transfection System (Invitrogen, Darmstadt, Germany) with standard settings (1000 V, 40 ms, 2 pulses) and 1.5 μg of siRNA.

### Infection of cells

For host cell infection, *Yersinia* cultures were grown overnight at 27°C, diluted 1:20 in fresh Luria-Bertani (LB) broth and grown for another 2 h at 37°C to induce activation of the type three secretion system. Bacteria were centrifuged, resuspended in ice-cold PBS containing 1mM MgCl_2_ and CaCl_2_ and added to cells grown in antibiotic free cell culture medium at a multiplicity of infection (MOI) of 50 for 90 min. In knockdown experiments cells were transfected with siRNA for 24–48 h before infection. Primary human macrophages were infected for 90 min and 6 h.

### Preparation of nuclear and cytosolic cell fractions

HEK293T cells were harvested by scraping after two washing steps with ice-cold PBS, resuspended in PBS containing digitonin (130 μg/ml; Sigma) and protease inhibitors (Complete; Roche Diagnostics, Mannheim, Germany) and incubated on ice for 5–15 min until the majority of the cells (> 98%) appeared to be lysed according to microscopic analysis. For determining protein phosphorylation, tris-buffered saline (TBS) containing phosphatase inhibitor (PhosSTOP, Roche Diagnostic, Mannheim, Germany) was used instead of PBS. Cell extracts were centrifuged at 2000 × g at 4°C for 10 min. The supernatant was saved as cytosolic fraction and pellets were resuspended in PBS containing 0.5% NP-40 and protease inhibitors followed by sonication to yield a homogenous nuclear fraction. Purity of nuclear and cytosolic fractions was assessed by virtual absence of Glyceraldehyde-3-phosphate-Dehydrogenase (GAPDH) and nuclear Lamin A/C, respectively, as tested by immunoblot.

### Leptomycin B treatment

Cells were serum starved for approximately 30 min and then incubated with 25 nM LMB (Sigma-Aldrich, Missouri, USA) for 3–4 h.

### Immunofluorescence staining and confocal microscopy

HeLa cells grown on glass coverslips (Marienfeld GmbH, Lauda-Königshafen, Germany) were fixed with 3.7% (v/v) formaldehyde in PBS for 5 min, permeabilized with 0.1% Triton X-100 in PBS for 5 min, blocked with 5% BSA in PBS for at least 15 min and incubated with a 1:100 dilution of primary antibody for 1 h followed by a 1:200 dilution of secondary antibody for 45 min. After three wash steps in PBS, coverslips were mounted in Mowiol (Calbiochem, Darmstadt, Germany). For nucleoporin staining (Mab414) cells were permeabilized with 40 μg/ml digitonin (Sigma) in HPEM buffer (30 mM HEPES, 2 mM MgCl2, 10 mM EGTA pH 7.4, 1 mM PMSF) for 2 min on ice before fixation [37]. Images were acquired with a confocal laser scanning microscope (Leica DMI 6000 with a Leica TCS SP5 AOBS confocal point scanner) equipped with a 63 x oil immersion HCX PL APO CS objective (NA 1.4–0.6). Acquisition was performed with Leica LAS AF software (Leica Microsystems, Wetzlar, Germany). Volocity 6 software (Improvision, Coventry, UK) was used for processing of images.

### Library preparation and high-throughput sequencing

Total RNA of human macrophages was isolated using the RNeasy extraction kit (Qiagen) according to manufacturer's instructions. mRNA was extracted using the NEBNext Poly(A) mRNA Magnetic Isolation module (New England Biolabs; NEB) and RNA-seq libraries were generated using the NEBNext Ultra RNA Library Prep Kit for Illumina (NEB) according to manufacturer's instructions. Size and quality of the libraries were assessed using a BioAnalyzer High Sensitivity Chip (Agilent). Diluted libraries were multiplex-sequenced on the Illumina HiSeq 2500 instrument (single read 51 bp run) with 39.1–55.8 million reads per sample.

Reads were aligned to the human reference assembly (GRCh38.97) using STAR [75]. FeatureCounts [76] was employed to obtain the number of reads mapping to each gene. Based on these counts, statistical analysis of differential expression was carried out with DESeq2 ([77], S9 Table). Sequence data for all 12 samples have been submitted to the European Nucleotide Archive (ENA) and will be publicly available at http://www.ebi.ac.uk/ena/data/view/PRJEB10086.

### IL-10 expression in macrophages by quantitative RT-PCR

Uninfected or infected primary human macrophages were subjected to RNA extraction using the RNeasy Mini Kit (Qiagen, Hilden, Germany). 500 ng to 2 μg of each RNA was reverse transcribed using iScript cDNA Synthesis Kit (Bio-Rad Hercules, California, USA) and subjected to RT-PCR reaction using the TaqMan Fast Advanced Master mix (Applied Biosystems, Carlsbad, California, USA) and gene specific primers. For determination of IL-10 mRNA levels the TagMan Gene Expression Assay for human IL-10 (Hs00961622_m1) was employed. As reference the TagMan Gene Expression Assay for GAPDH (Hs02758991_g1), TATA-box binding protein (TBP) (Hs00427620_m1) and beta-2-microglobulin (B2M) (Hs00187842_m1) was used (all from Thermo Fisher Scientific Waltham, Massachusetts, USA). PCR was performed using the LightCycler 480 Instrument (Roche Life Science) and data were analyzed according to manufacturer’s instruction (Roche LightCycler 480 software; Software release 1.5.1.62). Reference genes and external standards were employed for the relative quantification of IL-10 expression. GraphPad employing one-way Anova with Bonferroni´s post test was used for statistics.

### Western blot analysis and image quantification

Proteins were separated by SDS-PAGE and transferred to polyvinylidene difluoride (PVDF) membrane (Immobilion-P, Millipore, Schwalbach, Germany) by semi-dry blotting. The membrane was incubated in blocking solution (5% milk powder (w/v) in TBS supplemented with 0.01% Tween 20; TBS-T) at room temperature for 30 min, with primary antibodies at 4°C overnight, with secondary antibodies at room temperature for 2 h with extensive washing in TBS-T between steps. In GST-pulldown experiments PVDF membranes were blocked in TBS-T containing 25 mg BSA/ml instead of milk powder for 1 h followed by incubation with HisProbe-HRP conjugate (Thermo Scientific, Rockford, USA) at 4°C for 1 h. Antibody signals were visualized with chemiluminescence technology (Supersignal West Femto, Pierce Chemical, Rockford, USA) and captured on X-ray films (Fujifilm, Düsseldorf, Germany). For quantification of protein band intensity, films were scanned with a CanonScan 4400F (Canon, Tokio, Japan) resolution of 300–500 dpi and signals were analyzed using ImageJ analysis software Version 1.43u (National Institute of Health, NIH).

### Protein expression

*E*. *coli* BL21-AI harboring suitable expression vectors were grown to an optical density of 0.5–0.8 at 600 nm and protein expression was induced with 0.5 mM IPTG and 0.2% L-arabinose for 4 h. Purification steps included affinity chromatography on glutathione sepharose or Ni-NTA-Agarose, Tabacco Etch Virus (TEV)- or thrombin protease cleavage of the N-terminal 6x His- and GST-Tag, respectively, according to the manufacturer's instruction. Proteins were further purified by anion exchange chromatography using a UNO Q6 column (Biorad, Berkeley, USA) in an ÄKTAexplorer FPLC system (GE Healthcare, St Gilles, UK) according to the manufacturer's instruction.

### GST-YopM- and GST-myc-YopM pulldown

GST-YopM or GST (40 μg per sample) were incubated with 75 μl glutathione sepharose 4B beads (GE Healtcare) at 4°C for 2 h. After washing with 1 ml binding buffer (20 mM Tris pH 7.5, 500 mM NaCl, 0.5% NP40) the beads were incubated with lysate from *E*. *coli* BL21-AI expressing 6x CT-His fusion constructs of DDX3 at 4°C overnight followed by extensive washing in wash buffer (10 mM Tris pH 7.5, 150 mM NaCl, 0.1% NP40). For GST-myc-YopM pulldown HEK293T cells (6–8 x 10^6^) were cotransfected with expression vectors for 48 h, harvested in lysis-buffer (PBS, 1% Triton X-100, protease inhibitors) and lysed by three consecutive freeze/thaw cycles. Equal protein amounts of cell lysates were incubated with 20 μl of glutathione sepharose beads at 4°C overnight. Beads were washed extensively in ice cold lysis buffer and subjected to SDS-PAGE and immunoblot. Protein amounts were quantified by Bradford Protein Assay (Bio-Rad Laboratories GmbH, Munich, Germany).

### Tandem Affinity purification of SBP-CBP-tagged YopM

J774A.1 macrophages (approximately 4 x 10^8^) were infected with *Yersinia* strains translocating SBP-CBP-tagged YopM or with suitable control strains as described in Table 1. Precipitation of translocated SBP-CBP-tagged proteins was performed with the Interplay Tandem Affinity Purification kit (Agilent Technologies, Santa Clara, USA) according to the manufacturer’s instruction as described previously [20]. The CBP-binding Calmodulin beads were directly subjected to SDS-PAGE and immunoblot.

### Immunoprecipitation

Control- or transfected HEK293T cells (6–8 x 10^6^) were lysed in 1 ml of lysis buffer (PBS/ 1% NP40/ protease inhibitor cocktail), incubated with 1 μg rabbit polyclonal anti-DDX3 antibody (Bethyl Laboratories, Montgomery, USA) at 4°C for 2–3 h, washed extensively with lysis buffer and incubated with 20 μl Protein A/G Plus-Agarose (Santa Cruz Biotechnology) at 4°C overnight. In infected HEK293T cells, DDX3 was immunoprecipitated using the MultiMACS Protein G Kit (Miltenyi Biotec GmbH, Bergisch Gladbach, Germany). HEK293T cells (18–20 x 10^6^) were harvested and lysed in 1 ml of μMACS Lysis-Buffer (150 mM NaCl, 1% Triton X-100, 50 mM Tris-HCl, pH 8) according to the manufacturer’s instruction. Lysates were centrifuged at 13.000 x g, 4°C for 10 min and incubated with 2 μg (20 μl) of mouse monoclonal anti-DDX3 antibody (Santa Cruz Biotechnology) at 4°C for 15 min followed by incubation with 50 μl magnetic μMACS Protein G MicroBeads (Miltenyi Biotec GmbH) for 2 h. Immunoisolation using the μColumns and μMACS Separator were performed according to the manufacturer´s instruction. Immunoprecipitation of FLAG-tagged DDX3 and -PKN was performed with 20 μl Anti-FLAG-sepharose (Anti-FLAG M2 Affinity Gel, Sigma).

### Mass spectrometry

Mass spectrometry analysis of the host proteins coprecipitating with bacterially translocated YopM-SBP-CBP was previously described [20].

In short, peptide mass fingerprint data were determined on a MALDI-TOF mass spectrometer (REFLEX IV, Bruker Daltonics, Bremen, Germany) in reflector mode. Database searches were done with the Mascot search algorithm version 2.2 (Matrix Sciences, London, UK) using the following parameters: mass tolerance: 50 ppm; one missed tryptic cleavage allowed; fixed modification: carbamidomethyl cysteine; variable modification: monooxidized methionine; database searched: NCBI nr 20100116; searches limited to *mus musculus*.

### Analytical size exclusion experiments

One mg each of purified YopM from strain WA314 and DDX3 protein was loaded onto a Superdex 200 10/300 column (GE Healthcare, St Gilles, UK) equilibrated with gel filtration buffer (50 mM HEPES pH 7.5, 150 mM NaCl, 2mM DTT) and run on an ÄKTAexplorer FPLC system (GE Healthcare, St Gilles, UK). For analysis of the YopM:DDX3 complex, equimolar amounts of YopM and DDX3 were coincubated in gel filtration buffer at 4°C for 30 min. A 0.5 ml sample was loaded onto the column and run with 0.4 ml/min. Fractions of 1 ml were collected and analyzed by SDS-PAGE. Purified YopM from strains 8081 and YPIII were dialyzed in Slide-A-Lyser MINI Dialysis Units 3.500 MWCO (Thermo Scientific, Rockford, USA) against gel filtration buffer (50 mM HEPES pH 7.5, 250 mM NaCl) at 4°C for 2h. Sixty-seven μg of the proteins were loaded onto a Superdex 200 increase 3.2/300 column (GE Healthcare, St Gilles, UK) equilibrated with gel filtration buffer and run on SMART micropurification system (Pharmacia Biotech AB, Uppsala, Sweden). For analysis of the YopM:DDX3 complex, equimolar amounts of the proteins were coincubated in gel filtration buffer at room temperature for 15 min. A 50 μl sample was loaded onto the column and run with 40 μl/min.

### Microscale thermophoresis (MST)

DDX3_1–418 and DDX3_51–418 were fluorescently labelled using the Monolith Protein Labeling Kit RED-NHS (Nanotemper, Munich) following the manufacturer's protocol. Samples containing 16 serial 1:1 dilutions of YopM or YopM_34–481 producing concentration ranges from 224.000–6.85 nM or 278.000 to 8.48 nM, respectively, were prepared and each sample was incubated with 50 nM labeled DDX3 at room temperature for 30 min. Binding affinities were determined using a Monolith NT.115 (Nanotemper, Munich, Germany) as reviewed elsewhere [78]. All measurements were performed in 50 mM HEPES pH 7.5, 150 mM NaCl, 0.05% Tween, 0.5% bovine serum albumin at 50% LED and 20, 40 or 60% laser power using hydrophilic capillaries. Data analysis was performed using the Nanotemper analysis software and curves were fitted with Graph Pad Prism using nonlinear regression.

### Crystallization, data collection and structure determination of YopM

A fragment of YopM (amino acids 34–481) was purified as described above and adjusted to a protein concentration of 10.5 mg/ml in 50 mM HEPES pH 7.5. Preliminary crystallization experiments were carried out at 293 K in 96-well plates (NeXtal QIA1 μ-plates, Qiagen) using the following commercially available screening kits: PACT premier, Stura Footprint, Morpheus (Molecular Dimensions, UK) and Classic, ComPAS, JCSG+ Suite, AmSO_4_ (Qiagen) employing the sitting drop vapour diffusion method. Droplets of 1 μl (500 nl protein and 500 nl reservoir solution) were equilibrated against 50 μl reservoir solution. Initial crystals appeared after 3 days of incubation in several conditions. Condition PACT premier F11 (0.1 M Bis Tris propane pH 6.5, 0.2 M sodium citrate, 20% w/v PEG 3350) was further optimized to obtain crystals that were suitable for X-ray analysis. A 2 μl drop of protein solution was mixed with 2 μl of reservoir solution containing 0.05 M Bis Tris propane pH 6.5, 0.2 M sodium citrate, 24% w/v PEG 3350 and 15% glycerol using the sitting drop technique in a 24-well Linbro plate. Crystals grew to dimensions of 1 x 0,4 x 0,02 mm after 7 days. Diffraction data were collected to 3.2 Å resolution at Beamline P14 @ Petra III/EMBL/DESY (Deutsches Elektronen Synchrotron). Crystals were flash frozen in a stream of nitrogen at 100 K before applying radiation. Data were collected at a wavelength of 0.979570 using a PILATUS 6M detector, processed with iMOSFLM [79] and scaled with SCALA [80]. The crystal structure was solved by molecular replacement with MOLREP [81] using the structure of the LRRs of *Y*. *pestis* YopM (PDB entry 1jl5 [16]) as a search model. The structure was carefully built using a bootstrapping procedure involving multiple model building rounds interspersed using maximum likelihood refinement in REFMAC5 [82, 83] and the progress was monitored by a continuous decrease of the free R-value [84]. The program COOT was used for model building [85]. Solvent molecules were checked to confirm chemically reasonable positions where difference electron density exceeded a 3 σ level. For further analysis and refinement, programs from the CCP4 suite [83] were used and model building was carried out with COOT [85]. The final electron density maps were of very good quality and the models indicated good stereochemistry. The quality of the final model was assessed by PROCHECK [86]. The data collection and refinements statistics are summarized in S3 Table. The coordinates for the structures, as well as the experimental diffraction amplitudes have been deposited at the Protein Databank (http://www.rcsb.org) with entry codes 4OW2. For the analysis of the intermolecular interactions the webserver PDBePISA was used [34].

### Small angle X-ray scattering (SAXS)

Synchrotron X-ray scattering data of the protein solutions were collected at the EMBL beamline P12 on the PETRA III storage ring (DESY, Hamburg, Germany) [87]. Using a photon counting PILATUS 2M pixel detector (Dectris) at a sample-to-detector distance of 3.1 m and a wavelength of λ = 1.5 Å, the range of momentum transfer 0.004 < s < 0.45 Å^-1^ was covered (s = 4π sinθ/λ, where 2θ is the scattering angle). Solute concentrations ranging between 0.7 and 10.4 mg/ml were measured at 10°C. To monitor for the radiation damage, 20 successive 0.05- second exposures of protein solutions were compared and frames with statistically significant changes were discarded. The data were normalized to the intensity of the transmitted beam and radially averaged; the scattering of the buffer was subtracted and the difference curves were scaled for protein concentration. The low angle data measured at lower protein concentrations were extrapolated to infinite dilution and merged with the higher concentration data to yield the final composite scattering curves. The radius of gyration R_g_ was determined using the Guinier approximation assuming that at very small angles (s < 1.3/*R*_*g*_) the intensity is represented as I(s) = I(0) exp[(sR_g_)^2^/-3]. These primary data processing steps were done by the automated SAXS data analysis pipeline SASFLOW [88]. The pairwise distribution function p(r), the maximum intra-particle distance D_max_ along with another estimate of R_g_ were calculated using GNOM [89]. The molecular weight (MW) estimates were obtained from the Porod volume: for globular proteins Porod volumes in Å^3^ are about 1.6 times the MWs in Da.

A rigid body model of DDX3_51–418 was generated with coordinates of amino acid residues 168–418 taken from the structure of DDX3 (168–583, PDB entry 2I4I; [31]) and an ITASSER [90] model of residues 51–167 (C-score: -3.01). Modelling was performed with SASREF [91]. Starting from a tentative model, this program used simulated annealing to search for a non-overlapping interconnected configuration of the given structures fitting the experimental data. The ITASSER model was split into four rigid bodies in order to introduce some flexibility but it did not improve the fit. The YopM dimer was constructed by SASREF: given the structure of the monomer the program searched for a non-overlapping interconnected dimer that fits the experimental data. The model of the YopM_34-481/DDX3_51–418 complex was created using SASREF as well with the DDX3_51–418 model given as two rigid bodies as described above and the rigid dimer of YopM. Fits from all models to respective experimental data were recalculated with CRYSOL [92]. Given the atomic coordinates, the program minimizes discrepancy in the fit to the experimental intensity by adjusting the excluded volume of the particle and the contrast of the hydration layer. The graphical representation was done with Pymol (The PyMOL Molecular Graphics System, Version 1.7.4 Schrödinger).

The scattering data and the models are deposited in SASBDB [93], IDs:

YopM: SASDAU8 (http://www.sasbdb.org/data/SASDAU8/);
DDX3: SASDAV8 (http://www.sasbdb.org/data/SASDAV8/);
Complex: SASDAW8 (http://www.sasbdb.org/data/SASDAW8/).

### Statistical analysis

If not indicated otherwise statistical analyses were performed in GraphPad using two-way ANOVA with Bonferroni’s post test.

## Supporting Information

S1 Fig **A) Determination of the binding affinity between YopM and DDX3_1–418 or YopM and DDX3_51–418 by MST**. The dissociation constants (Kd) for the indicated interactions were determined by microscale thermophoresis (MST) as described in Methods. **B) Size exclusion chromatography calibration curves for (left panel) YopM, DDX3_1–418 and YopM:DDX3_1–418 complex and (right panel) YopM_34–481, DDX3_1–418 and YopM_34–481:DDX3_1–418 complex**. To obtain a calibration curve the ratio of the elution volume (Ve) of the indicated standard protein and the exclusion volume of the Superdex 200 10/300 column (Vo) was determined and plotted against the molecular weight of the respective protein (logarithmic scale). Standard proteins were thyroglobulin (670 kDa), γ-globulin (158 kDa), ovalbumin (44 kDa), myoglobin (17 kDa) and vitamin B12 (1.35 kDa). The apparent molecular weights of the color coded protein samples were calculated using the indicated equation and are indicated in Fig 2F or S1C Fig (below). **C) Size exclusion chromatography of YopM_34–481:DDX3_1–418 complex**. YopM_34–481, DDX3_1–418 or a 1:1 (molecular ratio) mixture of both proteins were individually subjected to size exclusion chromatography (color coded and super-imposed in the graph). Indicated fractions of the color coded chromatography runs were analyzed by SDS-PAGE. See S1B Fig for determination of molecular weight. **D) Size exclusion chromatography of YopM:DDX3_51–418 complexes using YopM isoforms from *Y*. *enterocolitica* 8081 and *Y*. *pseudotuberculosis* YPIII**. Full length YopM isoforms, DDX3_51–418 or 1:1 (molecular ratio) YopM/DDX3 mixtures were individually subjected to size exclusion chromatography (color coded and super-imposed in the graph).
(TIF)

S2 Fig Experimental fit of the SAXS data to the theoretical scattering curves from SASREF models. Experimental SAXS data (blue dots with error bars) and fits computed from the corresponding models (red lines) plotted as logarithm of the scattering intensity as a function of momentum transfer s = 4π sinθ/λ, where 2θ is the scattering angle and λ = 1.5 Å is the X-ray wavelength. A) DDX3_51–418; B) YopM_34–481 dimer; C) YopM_34–481:DDX3_51–418 complex. The curves are arbitrary displaced along the logarithmic axis for better visualization.
(TIF)

S3 Fig DDX3 is exported from the nucleus via CRM1. (Confocal micrographs) HeLa cells were treated without (Ctrl) or with 25 nM Leptomycin B (+ LMB) for 4 h and immunofluorescence stained using anti-DDX3 antibody. Scale bar, 20μm. (Bar graph) Mean fluorescence intensity (MFI) of nuclear and cytoplasmic DDX3 in control- (Ctrl) and LMB treated cells was determined. The MFI in Ctrl was set to 100%. Each bar represents mean ± SD of 100 cells from three different experiments; ***p<0.001.
(TIF)

S4 Fig C-terminally truncated YopM constructs unable to bind RSK do not increase RSK phosphorylation in the nucleus. Indicated myc-YopM constructs were expressed in HEK293T cells and anti-myc immunoprecipitated. Precipitates and whole cell lysates (WCL) were analyzed by Western blot using indicated antibodies.
(TIF)

S5 Fig YopM upregulates IL-10 expression in *Yersinia* infected human macrophages. Total RNA was isolated from primary human macrophages that were mock infected or infected with WA314 or WA314ΔYopM for 6 h. The RNA was subjected to quantitative RT-PCR using human IL-10 specific primers. IL-10 expression was normalized to expression of three housekeeper genes (GAPDH, TBP, B2M). For each condition triplicate samples of macrophages derived from seven different donors (Donor_1 to Donor_7) were investigated (data from Donor_1 to Donor_3 in Fig 6B).
(TIF)

S1 Table Peptide mass finger print analysis of host cell proteins coeluting with YopM-SBP-CBP. (PDF)

S2 Table Stereochemical and refinement parameters of the YopM _34–481 crystal. (PDF)

S3 Table SAXS data collection and derived parameters. (PDF)

S4 Table Intermolecular hydrogen bonds and salt bridges within the YopM_34–481 dimer. (PDF)

S5 Table DEGs in human macrophages infected with WA314ΔYopM vs. WA314 for 1.5 h. (PDF)

S6 Table KEGG pathway analysis of downregulated DEGs in WA314ΔYopM- vs. WA314 infected human macrophages at 1.5 h post infection. (PDF)

S7 Table KEGG pathway analysis of DEGs in the orange cluster of Fig 6. (PDF)

S8 Table Plasmids designed in this study. (PDF)

S9 Table Differential expression analysis of human macrophages uninfected (mock) or infected with WA314ΔYopM or WA314 for 1.5 h or 6 h. Duplicates (two different donors) of primary human macrophages were left uninfected or infected with *Y*. *enterocolitica* WA314 or -WA314ΔYopM for the indicated time periods. Total RNA was prepared from each sample and subjected to RNA-seq. Mean of the duplicates was formed (baseMean) and differentially expressed genes (DEGs) in different comparisions were determined. Statistical analysis of differential expression was carried out with DESeq2. Each sheet of the excel file contains the EntrezGene ID, the Associated Gene Name, gene description, log2-fold change and adjusted p-value (padj). P-values were calculated using DESeq2's implementation of the Wald test [77]. The Benjamini-Hochberg procedure was applied to obtain p-values adjusted for multiple testing.
(XLSX)
