# Supplementary figures and images for: Immunosuppressive Yersinia Effector YopM Binds DEAD Box Helicase DDX3 to Control Ribosomal S6 Kinase in the Nucleus of Host Cells
Source: PLoS Pathog. 2016 Jun 14;12(6):e1005660. doi: 10.1371/journal.ppat.1005660 (PMC4907486; doi:10.1371/journal.ppat.1005660)

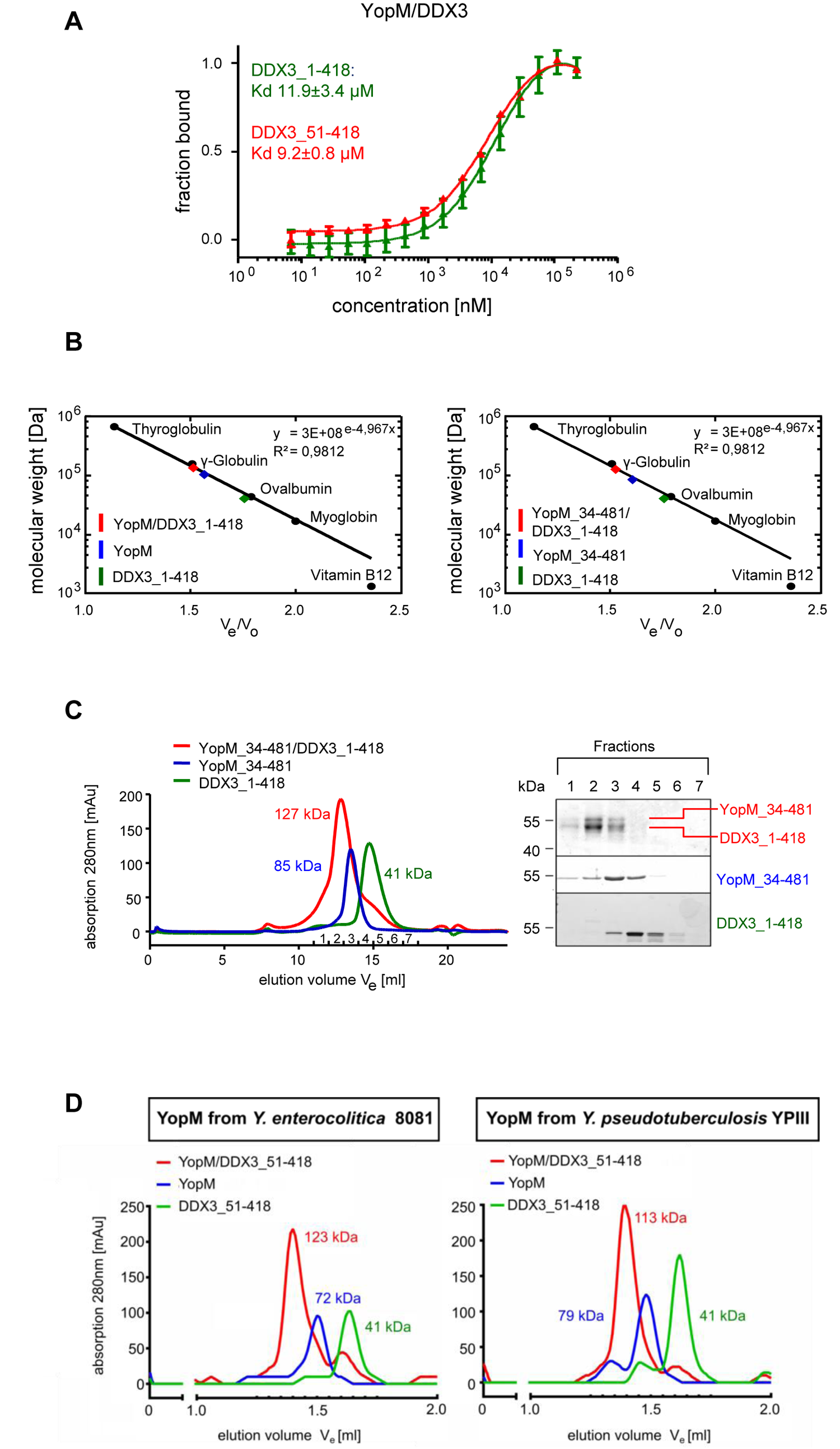

Supplement: S1 Fig — A) Determination of the binding affinity between YopM and DDX3_1–418 or YopM and DDX3_51–418 by MST. The dissociation constants (Kd) for the indicated interactions were determined by microscale thermophoresis (MST) as described in Methods. B) Size exclusion chromatography calibration curves for (left panel) YopM, DDX3_1–418 and YopM:DDX3_1–418 complex and (right panel) YopM_34–481, DDX3_1–418 and YopM_34–481:DDX3_1–418 complex. To obtain a calibration curve the ratio of the elution volume (Ve) of the indicated standard protein and the exclusion volume of the Superdex 200 10/300 column (Vo) was determined and plotted against the molecular weight of the respective protein (logarithmic scale). Standard proteins were thyroglobulin (670 kDa), γ-globulin (158 kDa), ovalbumin (44 kDa), myoglobin (17 kDa) and vitamin B12 (1.35 kDa). The apparent molecular weights of the color coded protein samples were calculated using the indicated equation and are indicated in Fig 2F or S1C Fig (below). C) Size exclusion chromatography of YopM_34–481:DDX3_1–418 complex. YopM_34–481, DDX3_1–418 or a 1:1 (molecular ratio) mixture of both proteins were individually subjected to size exclusion chromatography (color coded and super-imposed in the graph). Indicated fractions of the color coded chromatography runs were analyzed by SDS-PAGE. See S1B Fig for determination of molecular weight. D) Size exclusion chromatography of YopM:DDX3_51–418 complexes using YopM isoforms from Y. enterocolitica 8081 and Y. pseudotuberculosis YPIII. Full length YopM isoforms, DDX3_51–418 or 1:1 (molecular ratio) YopM/DDX3 mixtures were individually subjected to size exclusion chromatography (color coded and super-imposed in the graph). (TIF) [file ppat.1005660.s001.tif]

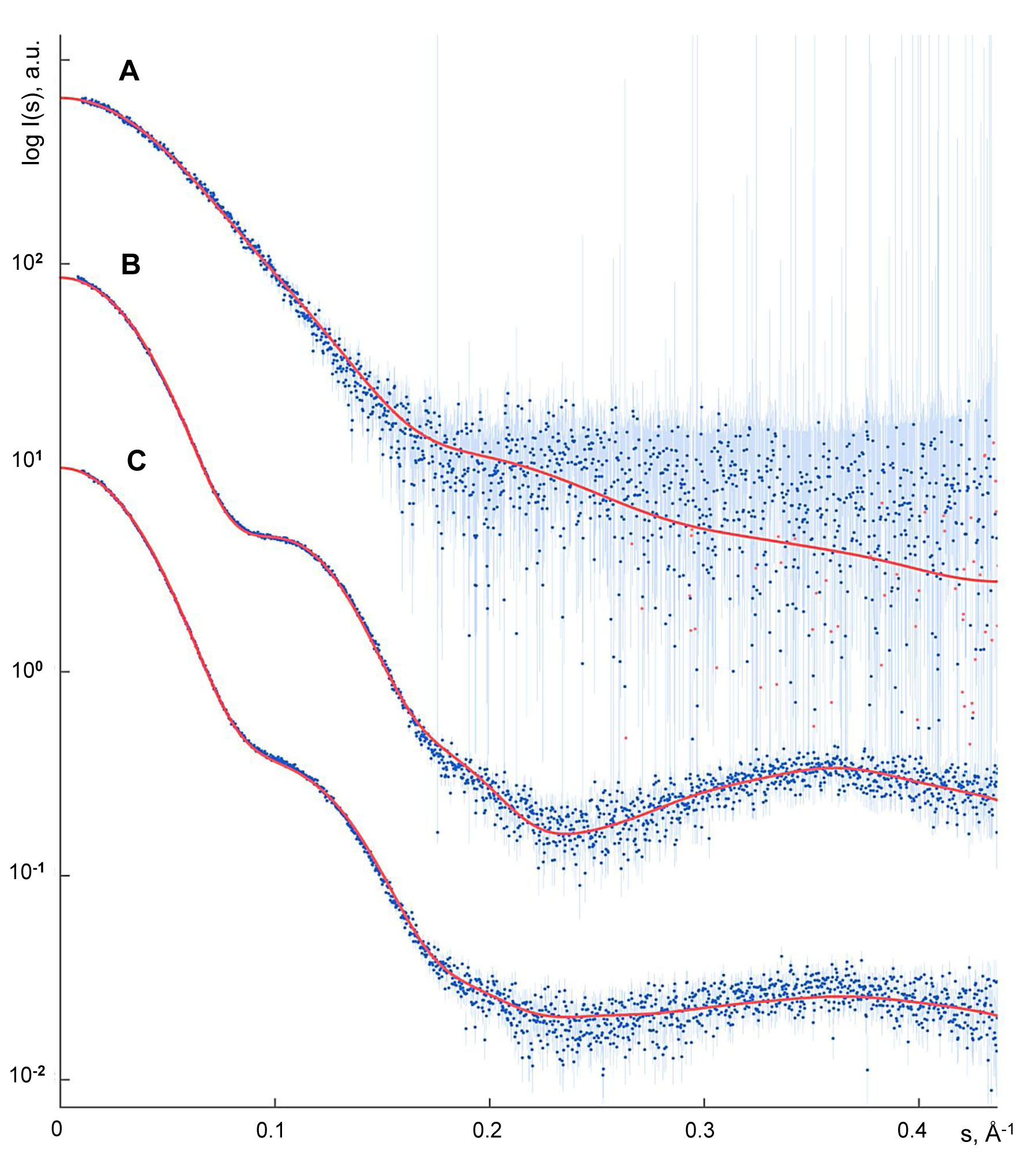

Supplement: S2 Fig — Experimental SAXS data (blue dots with error bars) and fits computed from the corresponding models (red lines) plotted as logarithm of the scattering intensity as a function of momentum transfer s = 4π sinθ/λ, where 2θ is the scattering angle and λ = 1.5 Å is the X-ray wavelength. A) DDX3_51–418; B) YopM_34–481 dimer; C) YopM_34–481:DDX3_51–418 complex. The curves are arbitrary displaced along the logarithmic axis for better visualization. (TIF) [file ppat.1005660.s002.tif]

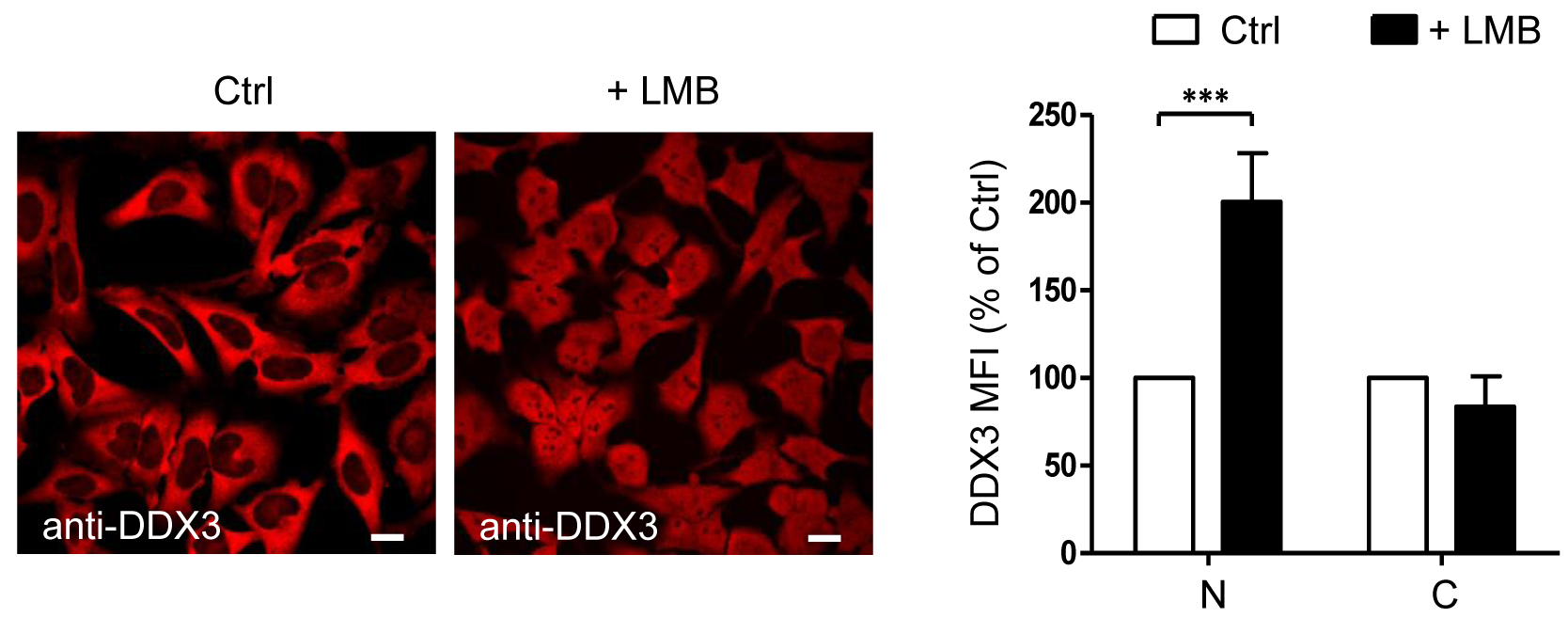

Supplement: S3 Fig — (Confocal micrographs) HeLa cells were treated without (Ctrl) or with 25 nM Leptomycin B (+ LMB) for 4 h and immunofluorescence stained using anti-DDX3 antibody. Scale bar, 20μm. (Bar graph) Mean fluorescence intensity (MFI) of nuclear and cytoplasmic DDX3 in control- (Ctrl) and LMB treated cells was determined. The MFI in Ctrl was set to 100%. Each bar represents mean ± SD of 100 cells from three different experiments; ***p<0.001. (TIF) [file ppat.1005660.s003.tif]

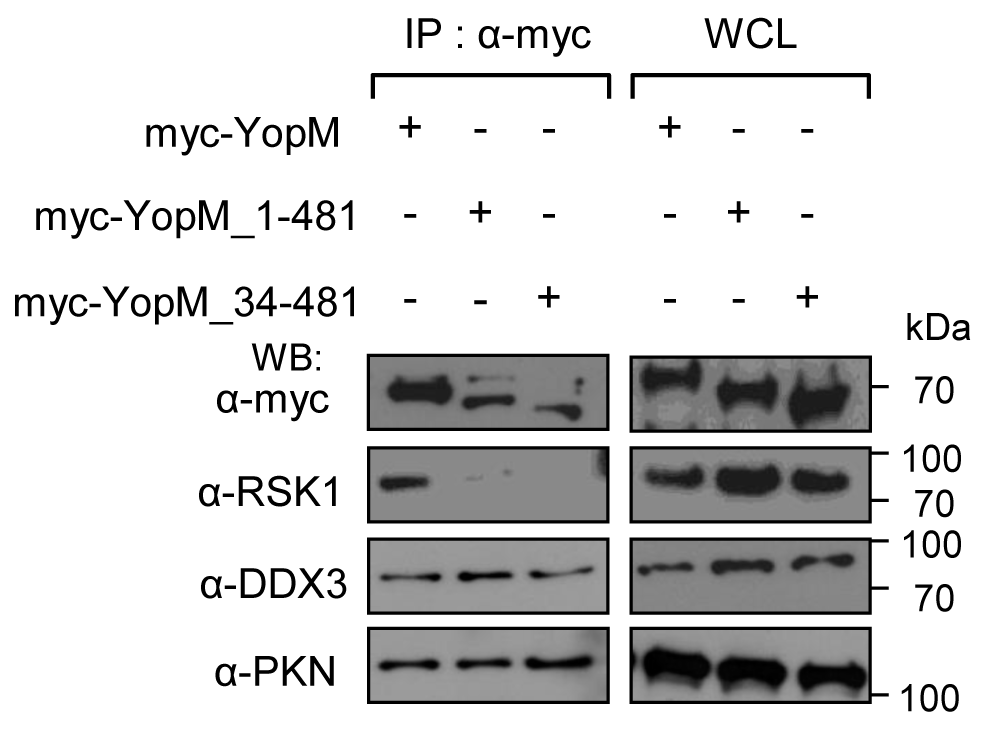

Supplement: S4 Fig — Indicated myc-YopM constructs were expressed in HEK293T cells and anti-myc immunoprecipitated. Precipitates and whole cell lysates (WCL) were analyzed by Western blot using indicated antibodies. (TIF) [file ppat.1005660.s004.tif]

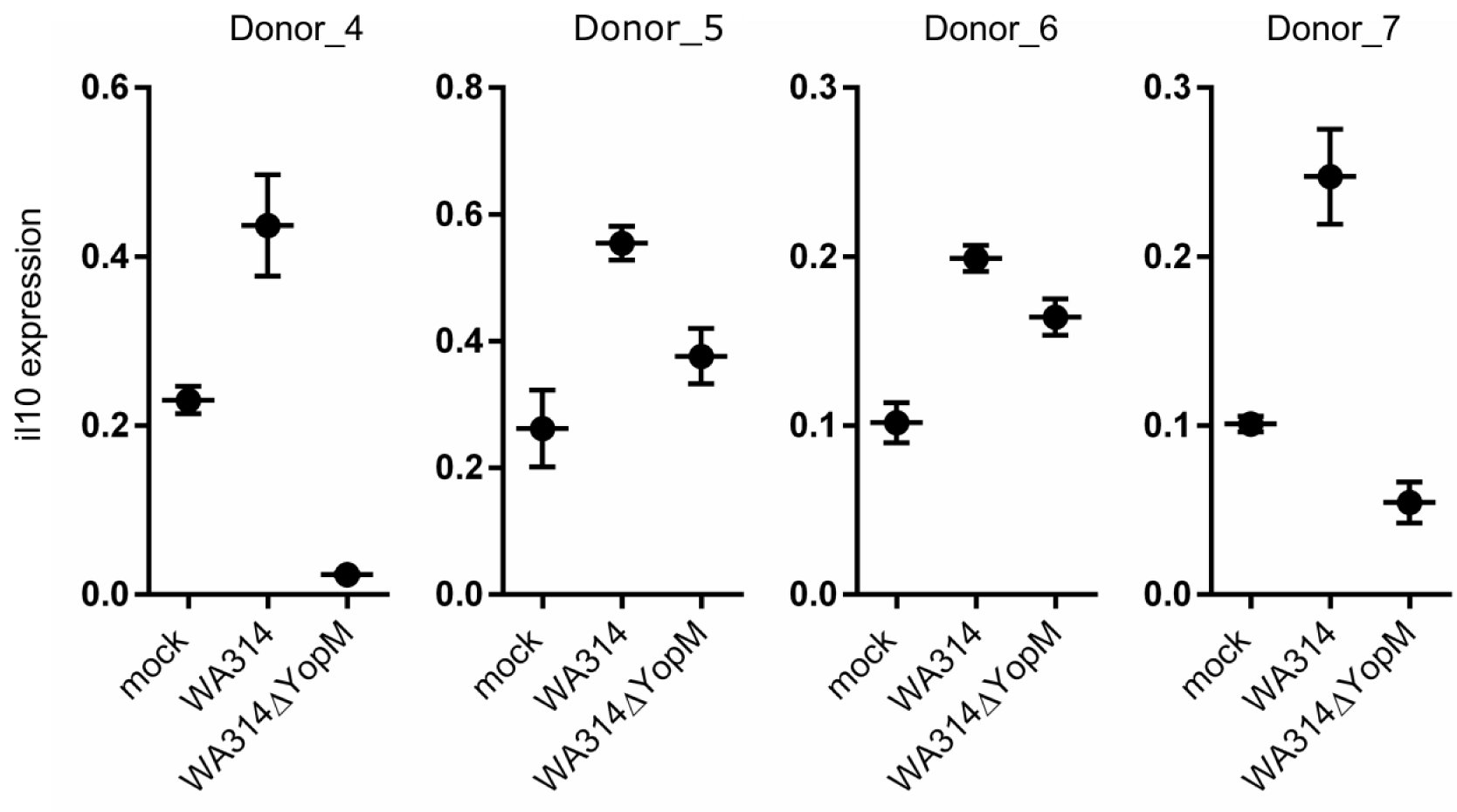

Supplement: S5 Fig — Total RNA was isolated from primary human macrophages that were mock infected or infected with WA314 or WA314ΔYopM for 6 h. The RNA was subjected to quantitative RT-PCR using human IL-10 specific primers. IL-10 expression was normalized to expression of three housekeeper genes (GAPDH, TBP, B2M). For each condition triplicate samples of macrophages derived from seven different donors (Donor_1 to Donor_7) were investigated (data from Donor_1 to Donor_3 in Fig 6B). (TIF) [file ppat.1005660.s005.tif]
