# Supplementary material for: Immunosuppressive Yersinia Effector YopM Binds DEAD Box Helicase DDX3 to Control Ribosomal S6 Kinase in the Nucleus of Host Cells
Source: PLoS Pathog. 2016 Jun 14;12(6):e1005660. doi: 10.1371/journal.ppat.1005660 (PMC4907486; doi:10.1371/journal.ppat.1005660)
Supplement: S1 Table — (PDF) [file ppat.1005660.s006.pdf]

S1 Table. Peptide mass finger print analysis of host cell proteins coeluting with YopM-SBP-CBP

protein	Mascot score	Expectation value	Peptide
DDX3X	99	1.7e-6	YIPPHLR; SDEDDWSKPLPPSER; LEQELFSGGNTGINFE; YTRPTPVQK; QYPISLVLAPTR; ELAVQIYEEAR; VRPCVVYGGAEIGQQI; GCHLLVATPGR; MLDMGFEPQIR; HTMMFSATFPK; EEALHQFR; SPILVATAVAAR; VGNLGLATSFFNER
DDX3Y	119	1.6e-8	YIPPHLR; GKPNYFSDR; SDEDDWSKPLPPSER; LEQELFSGGNTGINFE; YTRPTPVQK; QYPISLVLAPTR; ELAVQIYEEAR; VRPCVVYGGADTVQQI; GCHLLVATPGR; MLDMGFEPQIR; HTMMFSATFPK; YACTSIHGDR; EEALHQFR; KPILVATAVAAR

S1 Table: The final elution fraction of YopM-SBP-CBP tandem affinity purified from infected murine J774A.1 macrophages [1] was subjected to SDS-PAGE. Visible Coomassie-stained bands were excised, enzymatically digested and masses of the resulting peptides were subsequently determined on MALDI-TOF mass spectrometer. Data analysis of the obtained peptide mass fingerprints was performed with Mascot (http://www.matrixscience.com) obtaining indicated scores. The peptide-mass-fingerprint analysis significantly identified DDX3X or -Y ($p < 0.05$ at a score threshold of 55).
Using this methodology, RSK 1, 2 and PKN 1, 2 were confirmed as binding partners of YopM-SBP-CBP in previous work [1]. In the same experiments peptides matching the protein sequence of DEAD box RNA helicase 3 (DDX3) were identified. DDX3X and DDX3Y are encoded by the X- and Y-chromosome, respectively [2]. The peptide mass fingerprint analysis revealed indicated expectation values matching the DDX3X- or DDX3Y protein sequence. The lower the expectation value, the more significant the score.

References Supporting Information

1. Hentschke M, Berneking L, Belmar Campos C, Buck F, Ruckdeschel K, Aepfelbacher M. Yersinia virulence factor YopM induces sustained RSK activation by interfering with dephosphorylation. PloS one. 2010;5(10).

2. Sekiguchi T, Iida H, Fukumura J, Nishimoto T. Human DDX3Y, the Y-encoded isoform of RNA helicase DDX3, rescues a hamster temperature-sensitive ET24 mutant cell line with a DDX3X mutation. Experimental cell research. 2004;300(1):213-22.
