# Supplementary material for: Immunosuppressive Yersinia Effector YopM Binds DEAD Box Helicase DDX3 to Control Ribosomal S6 Kinase in the Nucleus of Host Cells
Source: PLoS Pathog. 2016 Jun 14;12(6):e1005660. doi: 10.1371/journal.ppat.1005660 (PMC4907486; doi:10.1371/journal.ppat.1005660)
Supplement: S2 Table — (PDF) [file ppat.1005660.s007.pdf]

S2 Table. Stereochemical and refinement parameters of the YopM _34-481 crystal

	**YopM_34-481**
Space group	C2
a (Å)	210.9
b (Å)	126.3
c (Å)	139.9
α (°)	90
β (°)	118.3
γ (°)	90
V_M (Å^3/Da)	4.1 assuming two dimers per asymmetric unit (AU)
Solvent content (%)	69.8
Resolution (Å)	40-3.2 (3.283 – 3.2)
No. of total refections	335667
Redundancy	6.6 (5.9)
Completeness of data (%)	99.6 (99.3)
Average I/sigma Intensity	11.9 (3.3)
No. of reflections used in refinement	50515
R_{merge} (%)[a]	9.1 (50.5)
R_{work} (%)[b]	18.1 (29.3)
R_{free} (%)[c]	20.1 (28.9)
Protein atoms	14167
Solvent atoms	59
Mean B value (Å^2):	101.7
Root mean square deviation:	
Bonds (Å)	0.006
Bond angels (°)	1.234

[a] R_{merge} is defined as $\sum_{hkl}\sum_i |I_i(hkl)-[I(hkl)]| / \sum_{hkl}\sum_i I_i(hkl)$, where $I_i(hkl)$ is the intensity of an individual measurement and $[I(hkl)]$ is the mean intensity from multiple observations of this refelction.

[b] $R_{work} = \sum|F_{obs}|-|F_{calc}| / \sum |F_{obs}|$, where F_{obs} and F_{calc} are observed and calculated structure factors.

[c] R_{free} was calculated using a randomly selected 5% of the reflections.
