# Supplementary material for: Immunosuppressive Yersinia Effector YopM Binds DEAD Box Helicase DDX3 to Control Ribosomal S6 Kinase in the Nucleus of Host Cells
Source: PLoS Pathog. 2016 Jun 14;12(6):e1005660. doi: 10.1371/journal.ppat.1005660 (PMC4907486; doi:10.1371/journal.ppat.1005660)
Supplement: S3 Table — (PDF) [file ppat.1005660.s008.pdf]

## S3 Table. SAXS data collection and derived parameters

| | | | |
|---|---|---|---|
| **Data collection parameters** | | | |
| Instrument | EMBL beamline P12 | | |
| Wavelength (Å) | 1.5 | | |
| S range ($Å^{-1}$) | 0.004–0.45 | | |
| Exposure time (s) | 20×0.05 | | |
| Concentration range (mg/ml) | 0.7–10.4 | | |
| Temperature (K) | 283 | | |
| **Structural parameters** | **DDX3_51-418** | **YopM_34-481** | **Complex** |
| $R_g$ (Å) (from Guinier) | 28±2 | 39±1 | 40±1 |
| $R_g$ (Å) (from p(r)) | 29±3 | 39±4 | 40±4 |
| $D_{max}$ (Å) | 120±12 | 115±12 | 150±15 |
| **Molecular weight determination** | | | |
| MW (kDa) from Porod volume | 50±5 | 96±10 | 119±12 |
| MW (kDa) from DAMMIF | 47±5 | 108±11 | 123±12 |
| Calculated MW (kDa) from sequence | 41.5 | 100.5 | 142.0 |
| **Software employed** | | | |
| Primary data reduction and processing | Automated SAXS data analysis pipeline SASFLOW | | |
| Ab initio analysis | DAMMIF, GASBOR | | |
| Rigid-body modelling | SASREF | | |
| Computation of model intensities | CRYSOL | | |
| 3-d graphics representation | Pymol | | |
