# Supplementary material for: Immunosuppressive Yersinia Effector YopM Binds DEAD Box Helicase DDX3 to Control Ribosomal S6 Kinase in the Nucleus of Host Cells
Source: PLoS Pathog. 2016 Jun 14;12(6):e1005660. doi: 10.1371/journal.ppat.1005660 (PMC4907486; doi:10.1371/journal.ppat.1005660)
Supplement: S4 Table — (PDF) [file ppat.1005660.s009.pdf]

S4 Table. Intermolecular hydrogen bonds and salt bridges within the YopM_34-481 dimer

	**Monomer 1**	**Dist. [Å]**	**Monomer 2**
1	ARG 479[NH2]	3.03	ASP 399[OD1]
2	ARG 479[NH2]	2.94	ASP 399[OD2]
3	ARG 479[NH1]	2.99	SER 401[OG]
4	ARG 479[NH2]	2.76	SER 401[OG]
5	ARG 479[NH2]	3.30	ASP 419[OD2]
6	ARG 361[NH2]	2.52	GLU 461[OE2]
7	ARG 342[NH1]	3.35	LEU 478[O]
8	ARG 402[NH2]	3.11	ASP 481[O]
9	ASP 399[OD1]	3.15	ARG 479[NH2]
10	ASP 399[OD2]	3.13	ARG 479[NH2]
11	SER 401[OG]	2.53	ARG 479[NH2]
12	ASP 419[OD2]	3.54	ARG 479[NH2]
13	ASP 422[OD2]	3.19	HIS 462[NE2]
14	GLU 461[OE2]	2.30	ARG 361[NH2]
15	MET 480[O]	3.52	ARG 361[NH1]
16	ASP 481[OD1]	3.35	ARG 402[NH1]
17	ASP 481[OD1]	3.27	ARG 402[NH2]
18	HIS 462[NE2]	3.30	ASP 422[OD2]
19	ARG 402[NE]	3.01	GLU 461[OE2]
20	LYS 362[NZ]	3.14	ASP 481[OD2]
21	GLU 461[OE2]	3.22	ARG 402[NH1]
