# Supplementary material for: Immunosuppressive Yersinia Effector YopM Binds DEAD Box Helicase DDX3 to Control Ribosomal S6 Kinase in the Nucleus of Host Cells
Source: PLoS Pathog. 2016 Jun 14;12(6):e1005660. doi: 10.1371/journal.ppat.1005660 (PMC4907486; doi:10.1371/journal.ppat.1005660)
Supplement: S5 Table — (PDF) [file ppat.1005660.s010.pdf]

S5 Table. DEGs in human macrophages infected with WA314ΔYopM vs. WA314 for 1.5 h

EntrezGene ID	Associated Gene Name	Description	log2-fold change	padj
9034	CCRL2	chemokine (C-C motif) receptor-like 2	-1.0339	0.00049
3280	HES1	hes family bHLH transcription factor 1	-1.7165	0.00292
1961	EGR4	early growth response 4	-2.111	0.00683
51561	IL23A	interleukin 23, alpha subunit p19	-3.04634	0.02647
165904	XIRP1	xin actin-binding repeat containing 1	-3.16580	0.02647
3586	IL10	interleukin 10	-1.84863	0.034

S5 Table: Duplicates (two different donors) of primary human macrophages were infected with *Y. enterocolitica* WA314 or -WA314ΔYopM for 1.5 h. Total RNA was prepared from each sample and subjected to RNA-seq. Mean of the duplicates was formed and differentially expressed genes (DEGs) (log2-fold change ≥ 1 and p ≤ 0.05) in WA314ΔYopM vs. WA314 infected macrophages were determined. padj; adjusted p-value.
