# Supplementary material for: Immunosuppressive Yersinia Effector YopM Binds DEAD Box Helicase DDX3 to Control Ribosomal S6 Kinase in the Nucleus of Host Cells
Source: PLoS Pathog. 2016 Jun 14;12(6):e1005660. doi: 10.1371/journal.ppat.1005660 (PMC4907486; doi:10.1371/journal.ppat.1005660)
Supplement: S6 Table — (PDF) [file ppat.1005660.s011.pdf]

S6 Table. KEGG pathway analysis of downregulated DEGs in WA314ΔYopM- vs. WA314 infected human macrophages at 1.5 h post infection

KEGG term (number of genes in the gene set)	EntrezGene IDs	Statistics
Cytokine-cytokine receptor interaction (3)	3586 51561 1236	C=244; E=0.12; R=25.96; adjP=0.0004
Jak-STAT signaling pathway (2)	3586 51561	C=139; E=0.07; R=30.38; adjP=0.0018

S6 Table: Kyoto Encyclopedia of Genes and Genomes (KEGG) pathway analysis of DEGs was performed using WebGestalt. The names/terms of the enriched KEGG pathways, the number of genes in the gene set of the respective pathway, the respective EntrezGeneIDs and the statistics for the enriched pathway are listed. C: number of reference genes in the category; E: expected number in the category; R: ratio of enrichment and adjP: p value adjusted by the multiple test adjustment.
