# Supplementary material for: Immunosuppressive Yersinia Effector YopM Binds DEAD Box Helicase DDX3 to Control Ribosomal S6 Kinase in the Nucleus of Host Cells
Source: PLoS Pathog. 2016 Jun 14;12(6):e1005660. doi: 10.1371/journal.ppat.1005660 (PMC4907486; doi:10.1371/journal.ppat.1005660)
Supplement: S7 Table — (PDF) [file ppat.1005660.s012.pdf]

**S7 Table. KEGG pathway analysis of DEGs in the orange cluster of Figure 6.**

| KEGG term (number of genes in the gene set) | EntrezGene IDs | Statistics |
|---|---|---|
| Cytokine-cytokine receptor interaction (11) | 7124 4283 3627 3592 6373 1233 6347 9966 3586 4049 515617 | C=265; E=1.10; R=10.02; adjP=4.18e-07 |
| African trypanosomiasis (5) | 7124 3620 3592 3586 3383 | C=35; E=0.14;R=34.49; adjP=5.41e-06 |
| Malaria (5) | 7124 3592 3586 6347 3383 | C=51; E=0.21; R=23.67; adjP=2.47e-05 |
| Jak-STAT signaling pathway (6) | 3592 1154 3586 51561 8835 9021 | C=155; E=0.64; R=9.35; adjP=0.0004 |
| Toll-like receptor signaling pathway (5) | 7124 4283 3627 3592 6373 | C=102; E=0.42; R=11.84; adjP=0.0004 |
| Staphylococcus aureus infection (4) | 3586 3075 3383 2358 | C=55; E=0.23;R=17.56; adjP=0.0005 |
| Allograft rejection (3) | 7124 3592 3586 | C=37; E=0.15; R=19.58; adjP=0.0024 |
| Rheumatoid arthritis (4) | 7124 51561 6347 3383 | C=91; E=0.38; R=10.61; adjP=0.0026 |
| Type II diabetes mellitus (3) | 7124 8835 9021 | C=48; E=0.20; R=15.09; adjP=0.0028 |
| Chagas disease/ American trypanosomiasis (4) | 7124 3592 3586 6347 | C=104; E=0.43; R=9.29; adjP=0.0028 |
| Amoebiasis (4) | 7124 3592 3586 5055 | C=106; E=0.44; R=9.11; adjP=0.0028 |
| Type I diabetes mellitus (3) | 7124 3592 4049 | C=43; E=0.18; R=16.85; adjP=0.0028 |
| Chemokine signaling pathway (5) | 4283 3627 6373 1233 6347 | C=189; E=0.78; R=6.39; adjP=0.0029 |
| NOD-like receptor signaling pathway (3) | 7124 6347 4210 | C=58; E=0.24; R=12.49; adjP=0.0044 |
| RIG-I-like receptor signaling pathway (3) | 7124 3627 3592 | C=71; E=0.29; R=10.20; adjP=0.0070 |
| Leishmaniasis (3) | 7124 3592 3586 | C=72; E=0.30; R=10.06; adjP=0.0070 |
| Asthma (2) | 7124 3586 | C=30; E=0.12; R=16.10; adjP=0.0136 |
| Glycine, serine and threonine metabolism (2) | 29968 26227 | C=32; E=0.13; R=15.09; adjP=0.014 |

**S7 Table:** For the orange cluster depicted in Fig 6, all enriched pathways with adjP $\leq$ 0.01 are shown. The names/terms of the enriched Kyoto Encyclopedia of Genes and Genomes (KEGG) pathways, the number of genes in the gene set of the respective pathway, the respective EntrezGene IDs and the statistics for the enriched pathway are listed. DEGs: differentially expressed genes; C: number of reference genes in the category; E: expected number in the category; R: ratio of enrichment and adjP: p value adjusted by the multiple test adjustment.
