# Supplementary material for: Immunosuppressive Yersinia Effector YopM Binds DEAD Box Helicase DDX3 to Control Ribosomal S6 Kinase in the Nucleus of Host Cells
Source: PLoS Pathog. 2016 Jun 14;12(6):e1005660. doi: 10.1371/journal.ppat.1005660 (PMC4907486; doi:10.1371/journal.ppat.1005660)
Supplement: S8 Table — (PDF) [file ppat.1005660.s013.pdf]

S8 Table. Plasmids designed in this study

Vector	Insert	Primer	Resulting Plasmid
pGEX-4T2 (GE-Healthcare, Munich, Germany),	*yopM* full length (aa1-506)	YopM-for 5´-CTGGGATCCTTT ATAAATCCAAGA-3´ pGEX-YopM-rev 5´-GCCGAATTCCTACTCAAA AACATCATC-3	pGEX-4T2-YopM
pET302 (Thermo fisher scientific Waltham, USA, 6x-N-terminal (NT)- HIS tag	*yopM* full length (aa1-506) and recognition site for tobacco etch virus protease (TEV)	tev-YopM-for 5´-CGTGAATTCGGAGAATCT TTATTTTCAGGGCTTTATA AATCCAAGAAATGTA-3´tev-YopM-rev 5´ATTCGGATCCCTACTCA AAAACATCATCTTCAAG-3´	6xNT-His-TEV-YopM
pET302, 6x-NT- HIS tag	*yopM* aa34-481 and TEV- recognition site	tev-YopM_34-for 5´-CGTGAATTCGGAGAATCT TTATTTTCAGGGCAAATC TAAGACTGAATATTATAA TGCA-3´tev-YopM_34-481-rev 5´-ATTCGGATCCTTAGTCCA TCCGAAGATCTTCCAC -3´	6xNT-His-TEV-YopM_34-481
pCS2+MT (XB-VEC12442480), [1], myc tag	*yopM* full length (aa1-506)	YopM-NCOI-for 5´-ACCATGGTTATGTTTATA AATCCAAG-3´ YopM-XHOI-rev 5´-GTCAAAGCTTATGTATGG TTTTGTGGTCAATGAA-3´	myc-YopM
pCS2+MT, myc tag	*yopM* (aa1-481)	YopM-NCOI-for 5´-ACCATGGTTATGTTTATA AATCCAAG-3 YopM_481-XHOI-rev 5´-GTCACTCGAGCTAGTCCA TCCGAAGATC-3´	myc-YopM_1-481
pEBG2T, [3] GST-tag	*yopM* full length (aa1-506)	derived from myc-YopM and ligated into pEBG2T via restriction-sites BAMHI and NOTI	myc-GST-YopM
pET303 (Thermo fisher scientific, Waltham, USA), 6x-C-termnial (CT)- HIS tag	*ddx3* full length aa1-662	DDX3-for 5´-GAGGTCTAGAATGAGTCA TGTGGCAGTGG-3´ DDX3-rev 5´-TACCCTCGAGGTTACCCC ACCAGTC-3´	His-DDX3

pET303, 6x-C-CT- HIS tag	*ddx3* (aa1-418)	DDX3-for 5´-<br>GAGGTCTAGAATGAGTCA<br>TGTGGCAGTGG-3´<br>DDX3 _418-rev 5´-<br>TACCCTCGAGTTTCTGTGT<br>GA TGTTTTC-3´.	His-DDX3_1-418
pET303, 6x-CT- HIS tag	*ddx3* (aa51-418)	DDX3_51for 5´- GAGGTCT<br>AGAATGGGTTTCTACGAT<br>AAAGAC-3´<br>DDX3 _418-rev 5´-<br>TACCCTCGAGTTTCTGTGT<br>GA TGTTTTC-3´.	His-DDX3_51-418
pET303, 6x-CT- HIS tag	*ddx3* (aa101-418)	DDX3_101-for 5´-<br>GAGGTCTAGAATGCGGAG<br>TGATTACGATGGC-3´<br>DDX3 _418-rev 5´-<br>TACCCTCGAGTTTCTGTGT<br>GA TGTTTTC-3´.	His-DDX3_101-418
pET303, 6x-CT- HIS tag	*ddx3* (aa168-418)	DDX3_168-for 5´-<br>GAGGTCTAGAATGGTT<br>GAGGCAACAGGCAAC-3´<br>DDX3 _418-rev 5´-<br>TACCCTCGAGTTTCTGTGT<br>GA TGTTTTC-3´.	DDX3_168-418
pET303, 6x-CT- HIS tag	*ddx3* (aa201-418)	DDX3_201-for 5´-<br>GAGGTCTAGAATGACTCG<br>CCCAACTCCAGTG-3´<br>DDX3 _418-rev 5´-<br>TACCCTCGAGTTTCTGTGT<br>GA TGTTTTC-3´.	His-DDX3_201-418
pCDNA 3.1(+) (Thermo fisher scientific Waltham, USA)	*ddx3* full length (aa1-662)	DDX3-HINDIII-for 5´-CA<br>AGCTTATGAGTCATGTGG<br>CAGTGGAAAAT-3´<br>DDX3_662-HA-BAMHI-rev<br>5´-AGGATCCTCAAGCGT<br>AATCTGGAACATCGTATG<br>GGTACATGTTACCCCACC<br>AGTCAACC-3´	DDX3-HA
pCDNA 3.1(+)	*ddx3* (aa1-418)	DDX3-HINDIII-for 5´-CA<br>AGCTTATGAGTCATGTGG<br>CAGTGGAAAAT-3´<br>DDX3_1-418-HA-BAMHI-<br>rev 5´GGATCCTCAAGCGTA<br>ATCTGGAACATCGTATGG<br>GTACATTTTCTGTGTGATG<br>TTTTCAGAGGT-3	DDX3-HA aa1-418
pCDNA 3.1(+)	*ddx3* aa418-662	DDX3_418-602-for 5´-<br>CAAGCTTACCATGGTAGT<br>TTGGGTGGAAGA-3´<br>DDX3_662-HA-BAMHI-rev<br>5´-AGGATCCTCAAGCGT<br>AATCTGGAACATCGTATG<br>GGTACATGTTACCCCACC<br>AGTCAACC-3´	DDX3-HA aa418-662

pCDNA 3.1(+)	*ddx3* full length aa1-662	DDX3-for 5´-CAAGCTTACCATGAGTCATGTGGCAGTG-3´ DDX3-flag-BAMHI-rev 5´-AGGATCCTCACTTATCGTCGTCATCCTTGTAATCCATGTTACCCCACCAGTCAACC-3´	DDX3-flag
pCDNA 3.1(+)	*ddx3* full length aa1-662 with defect in ATPase- Helicase-activity obtained by mutation of aa K230E	DDX3_K230E-for 5´-GCCCAAACAGGGTCTGGAGAGACTGCAGCATTTCTGT TT-3´ DDX3_K230E-rev 5´-AACAGAAATGCTGCTGCAGTCTCTCCAG ACCCTGTTTGGGC -3´	DDX3K230E-HA
pCDNA 3.1(+)	*ddx3* full-length aa1-662 with two silent mutations resulting in resistance to DDX3 against siRNA no 3	siRNA3-DDX3-mutKit-for 5´-ATCATGGGAAACAT CGAGCTTACACGTTATACTCGCCCA-3´ siRNA3-DDX3-mutKit-rev :5´TGGGCGAGTATAACGTGTAAGCTCGATGTTTCCCATGAT-3´	rDDX3-HA
pACYC 184 [4]	*yopM* (aa1-481)	YopM_481-XHOI-rev 5´-GTCACTCGAGCTAGTCCATCCGAAGATC-3´ YopM –F-hind 5´-GTCAAAGCTTATGTATGGTTTTGTTTGCAATGAA-3´	pACYC 184 _YopM_1-481

References Supplemental Information

1. Hentschke M, Berneking L, Belmar Campos C, Buck F, Ruckdeschel K, Aepfelbacher M. Yersinia virulence factor YopM induces sustained RSK activation by interfering with dephosphorylation. PloS one. 2010;5(10).

3. Dettori R, Sonzogni S, Meyer L, Lopez-Garcia LA, Morrice NA, Zeuzem S, et al. Regulation of the interaction between protein kinase C-related protein kinase 2 (PRK2) and its upstream kinase, 3-phosphoinositide-dependent protein kinase 1 (PDK1). The Journal of biological chemistry. 2009;284(44):30318-27.

4. Chang AC, Cohen SN. Construction and characterization of amplifiable multicopy DNA cloning vehicles derived from the P15A cryptic miniplasmid. J Bacteriol. 1978;134(3):1141-56.
